# Supplementary material for: Evolution of communication signals and information during species radiation
Source: Nat Commun. 2020 Oct 2;11:4970. doi: 10.1038/s41467-020-18772-3 (PMC7532446; doi:10.1038/s41467-020-18772-3)
Supplement: Supplementary file 1 — Supplementary Information [file 41467_2020_18772_MOESM1_ESM.pdf]

SUPPLEMENTARY INFORMATION

Evolution of communication signals and information during species radiation

Garcia et al.

Supplementary Figs. 1-15

Supplementary Tables 1-13

Supplementary references

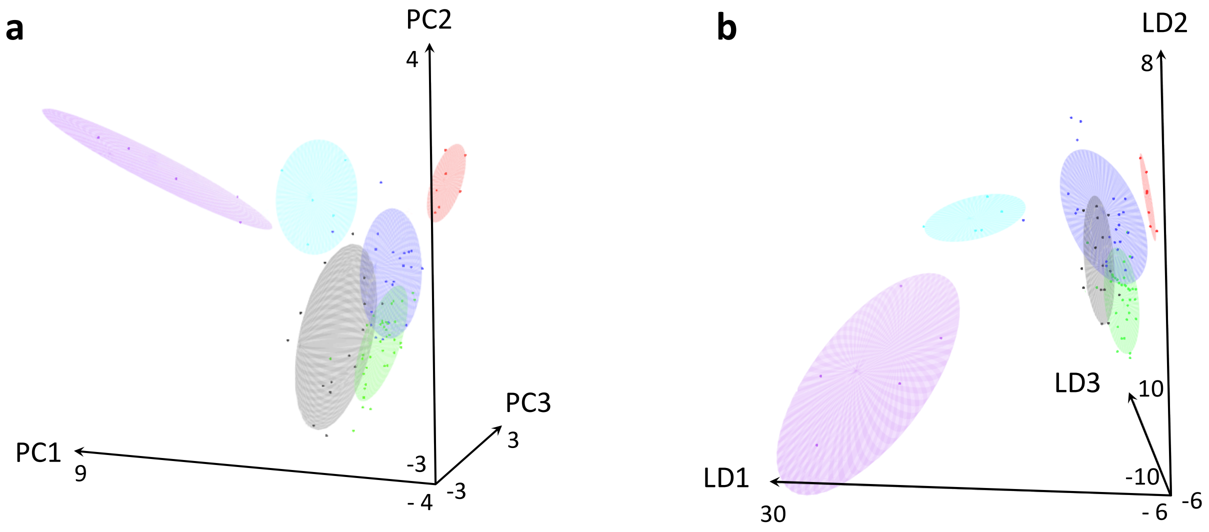

Supplementary Fig. 1. Embedding of the six drumming types in a 3D acoustic space, built either **a)** from a Principal Component Analysis based on the 22 acoustic variables used to characterize drumming signals, or **b)** from a Discriminant Function Analysis carried out using these Principal Components (see Methods for details). Each dot represents one woodpecker species. The first three Principal Components **(1a)** explained respectively 29, 14 and 11% of the variance in drumming acoustic structure (total = 54.5%, Supplementary Table 11). The first three Linear Discriminants **(2b)** explained respectively 58, 17 and 14% of the variance between species-specific drumming acoustic structure (total = 89%, Supplementary Table 12).

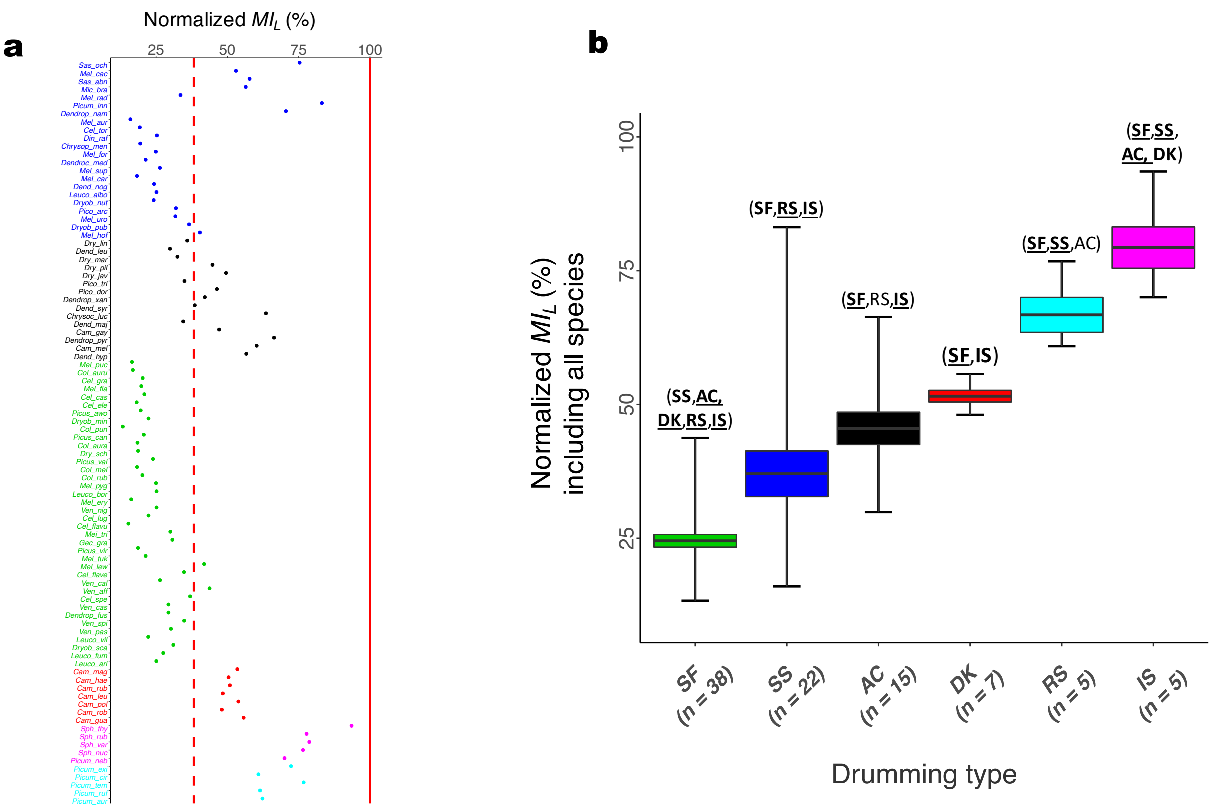

Supplementary Fig. 2. **a**, Normalized amount of information (i.e. normalized local mutual information values = MI_L) in each woodpecker species' drumming. The dashed red line indicates the averaged normalized mutual information across all species (i.e. the overall mutual information; n=92). The solid red line indicates the ceiling value of averaged normalized mutual information. **b**, Normalized MI_L for each drumming type (box plots denote mean ± SE (boxes) and min/max values (whiskers)). The number of species drumming with a given type is indicated in brackets. Tests and significance levels are indicated as follows: (SF) significant difference with SF; (SS) significant difference with SS; (AC) significant difference with AC; (DK) significant difference with DK; (RS) significant difference with RS; (IS) significant difference with IS. Abbreviations without formatting indicate $P < 0.05$; abbreviations in bold indicate $P < 0.005$; abbreviations in bold and underscored indicate $P < 0.001$; P values are adjusted for multiple comparisons between groups (one-way ANOVA, Tukey post hoc test).

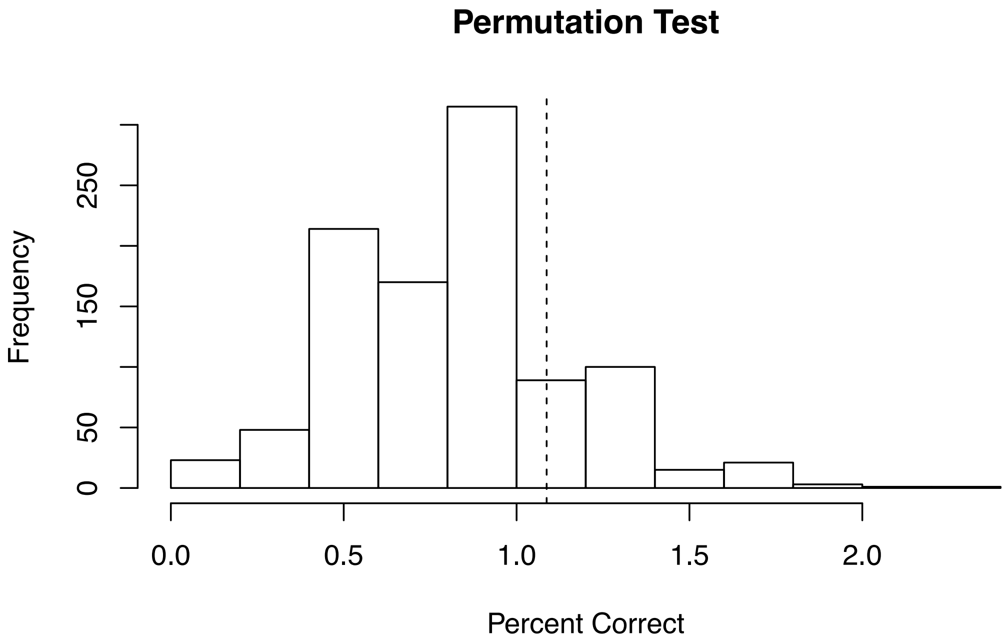

Supplementary Fig. 3. Histogram of percent correct classifications for 1000 permutations runs of a permuted DFA (pDFA). This permutation test shows that the classification rate of 16.5% produced by the DFA is highly significant ($p < 0.001$; dashed line is the 'chance' level = 1/ number of species = 1.09%, and the actual value of 16.5% is far off the chart).

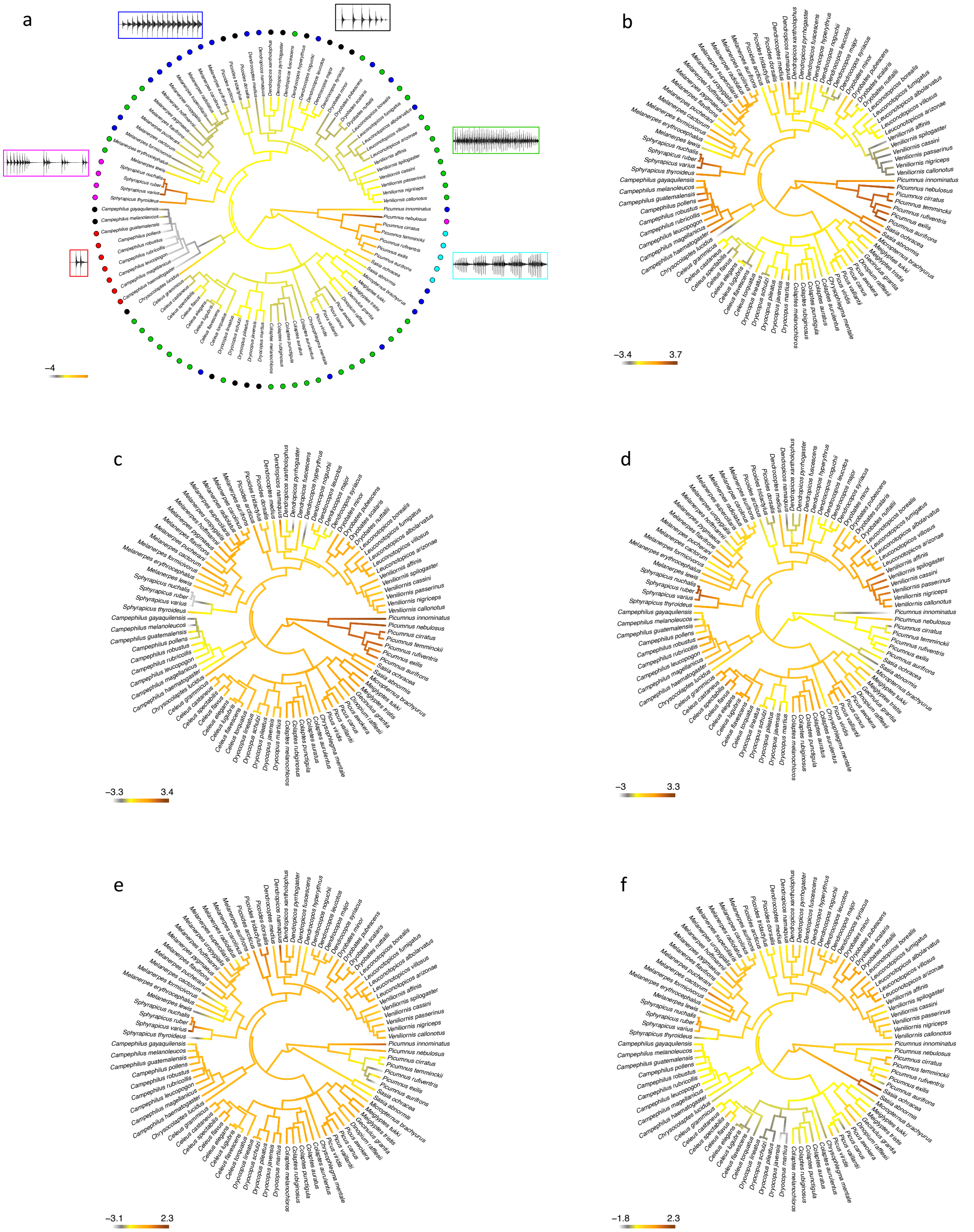

Supplementary Fig. 4. Ancestral state reconstruction of drumming acoustic structure (represented by PC1-PC6, respectively in panels a-f) along the woodpeckers' phylogenetic tree. Color palette of the tree branches corresponds to variation in PCs. In panel a, color dots around the tree in panel **a** illustrate the distribution of drumming types among living species. Similar signalling types are often shared among closely related species (Pagel's lambda = 0.925, P < 0.0001). All six reconstructions showed a high and significant phylogenetic signal (Supplementary Table 2).

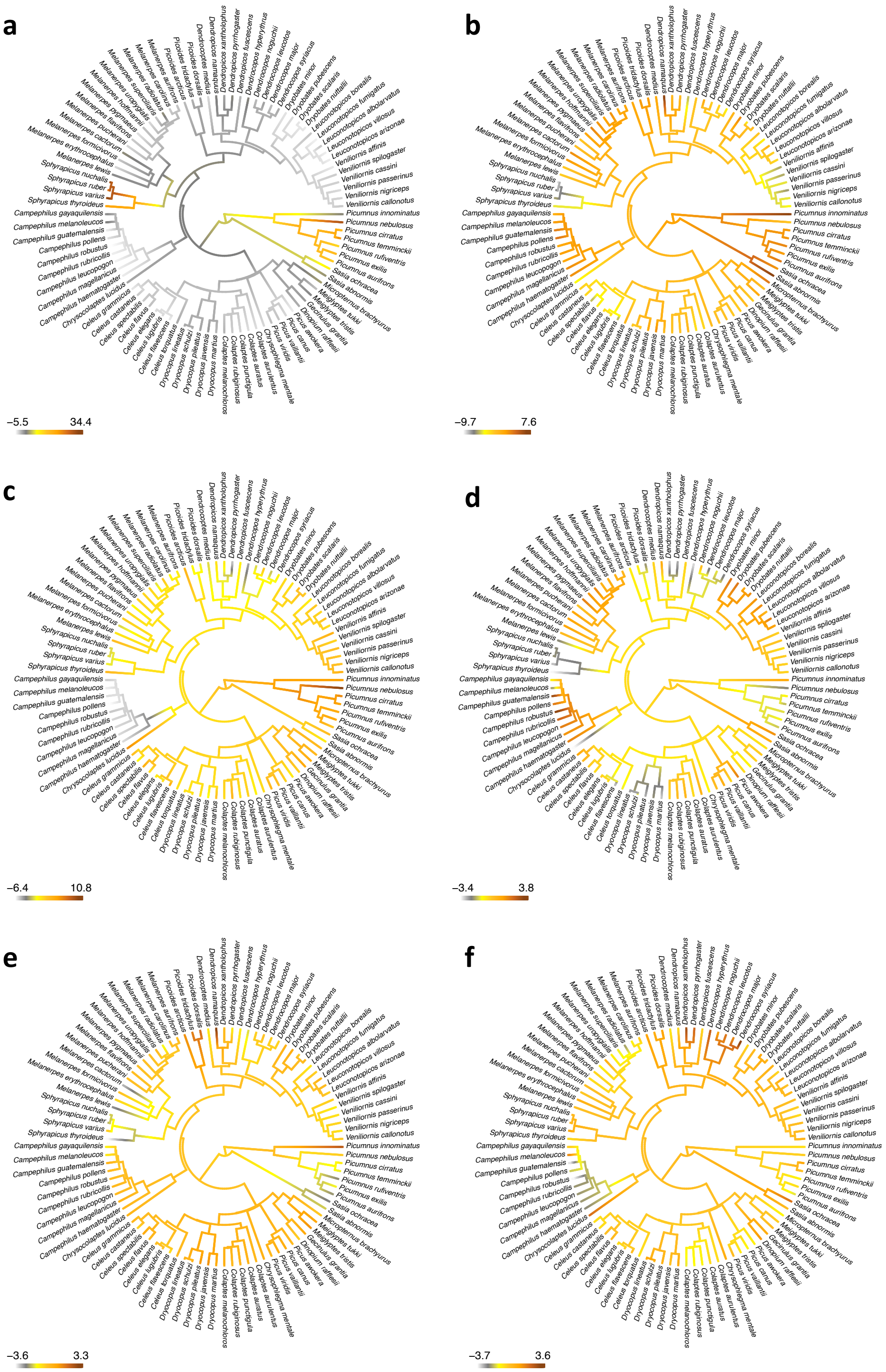

Supplementary Fig. 5. Ancestral state reconstruction of drumming acoustic structure (represented by LD1-LD6, respectively in panels a-f) along the woodpeckers' phylogenetic tree. Color palette of the tree branches corresponds to variation in LDs. All six reconstructions showed a high and significant phylogenetic signal (Supplementary Table 2).

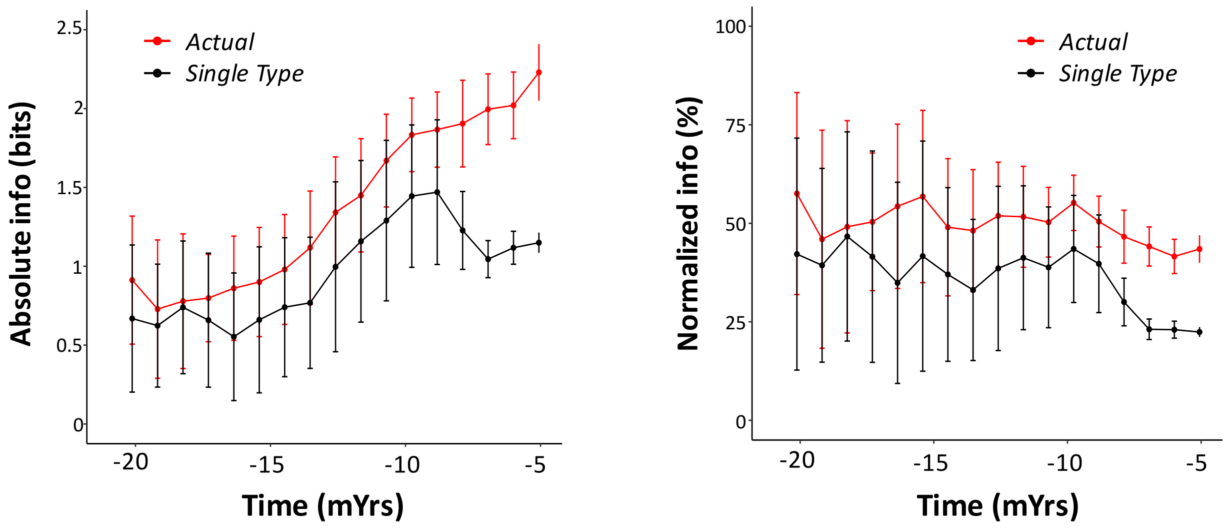

Supplementary Fig. 6. Reconstruction of absolute (left panel) and normalized (i.e. divided by the number of species on which information calculation is based; right panel) mutual information content. Calculation of mutual information follows the reconstruction of drumming types along the woodpeckers' phylogenetic tree. The distribution of drumming types at a given time in the tree is interpolated from drumming type probability distributions at known ancestral nodes. Mutual information values are calculated either following the reconstruction of known drumming types for each species ('*Actual*'; red lines on both panels), or by forcing the maintenance of a unique drumming type over time – a 'NULL' evolutionary scenario ('*Single Type*'; black lines on both panels). For the later scenario, we estimated the ancestral mutual information obtained using the drumming signals in the extant species using the same methodology than for the *Actual* reconstruction but sampling from species that used the same drumming types (*Single Type*). Each reconstruction was based on n = 30 simulations and error bars show ± 1 SD of mean information values obtained by resampling. The evolution of novel drumming types led to significantly higher mutual information values, in particular when the number of species increases (left panel), and allowed a higher level of species discrimination (represented by stable but higher normalized mutual information than the stable but lower level resulting from the NULL scenario – right panel). Note, we could only perform this simulation for up to 5 M years ago because the simulation involves sampling from extant species sharing the same drumming type. Beyond 5 Myrs, the number of species sharing a single drumming type is smaller than the number of ancestral species in the clade (as determined by the phylogenetic tree).

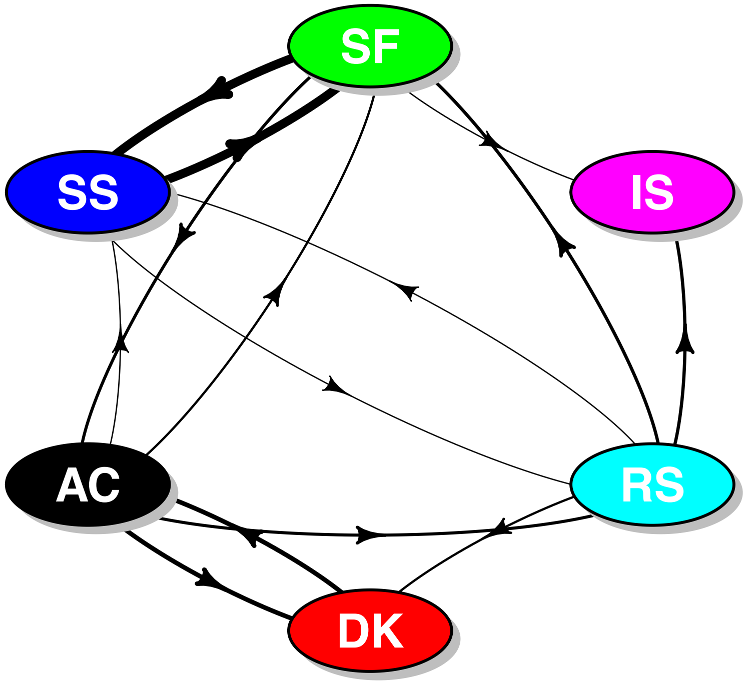

Supplementary Fig. 7. Transition probability diagram between drumming types. Line width represents the relative probability value of evolutionary transitions.

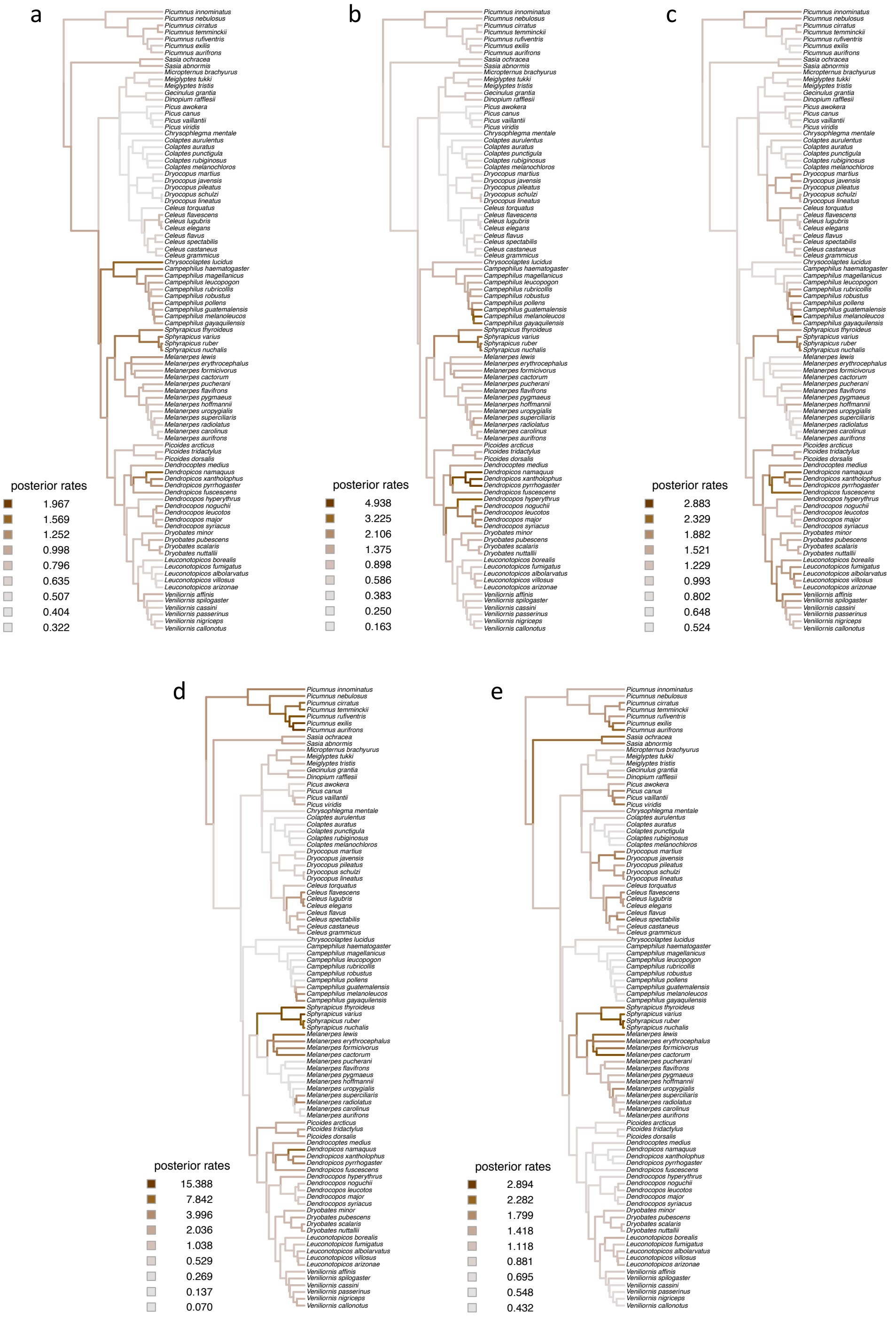

Supplementary Fig. 8. Similar to main text's Figure 4a, reconstruction of the evolutionary rates for drumming structure (PC2-PC6, respectively in panels a-e) along the woodpeckers' phylogeny ($n = 92$ species). Rates are standardized (variables divided by their standard deviation before model computation) and are represented by a color-gradient (see scales in the Figure).

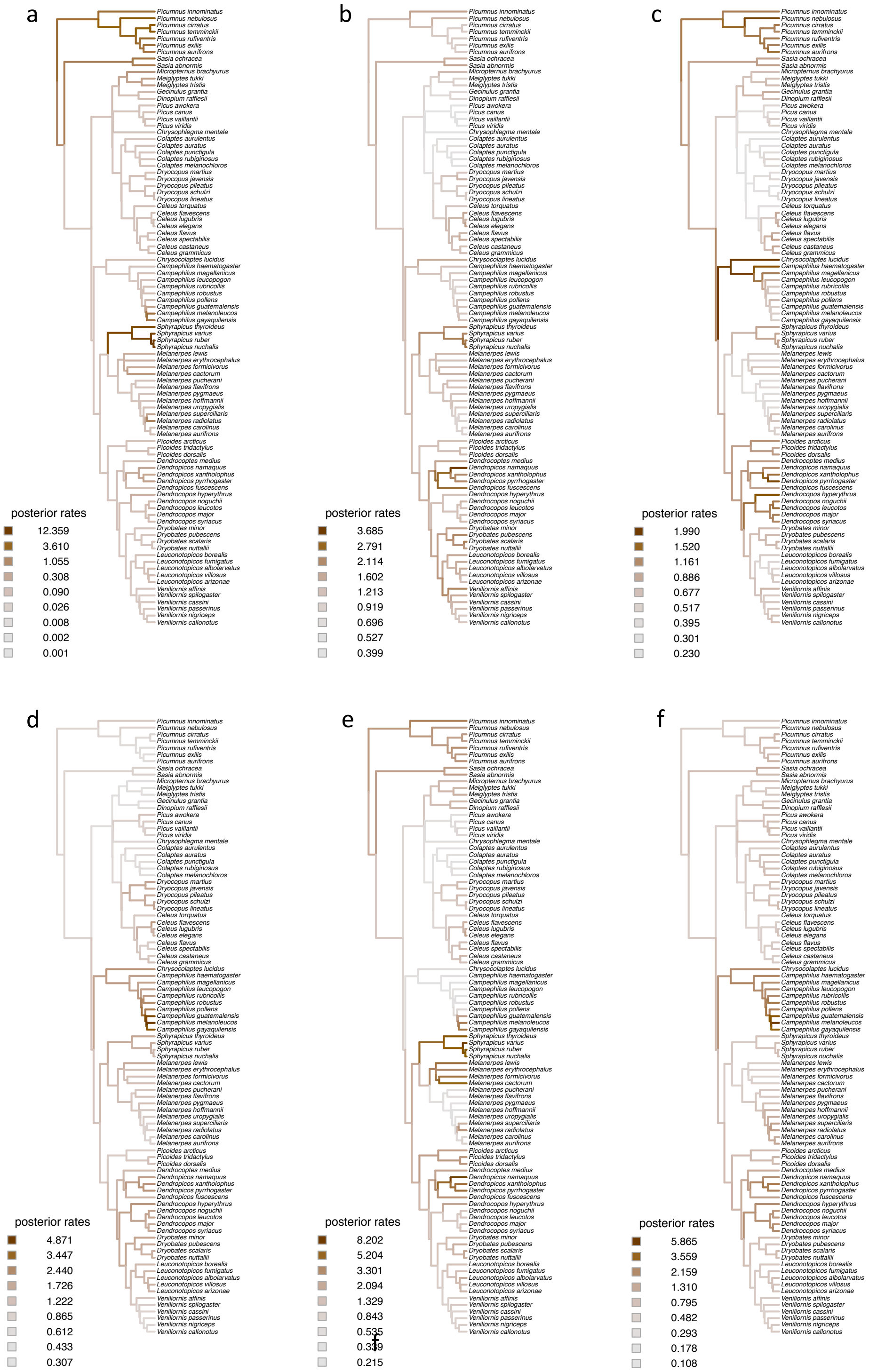

Supplementary Fig. 9. Similar to main text's Figure 4a, reconstruction of the evolutionary rates for drumming structure (using LD1-LD6, respectively in panels a-f) along the woodpeckers' phylogeny (*n* = 92 species). As with PCs (Figure 4a; Supplementary Fig. 8), rates are standardized (variables divided by their standard deviation before model computation) and are represented by a color-gradient (see scales in this Figure).

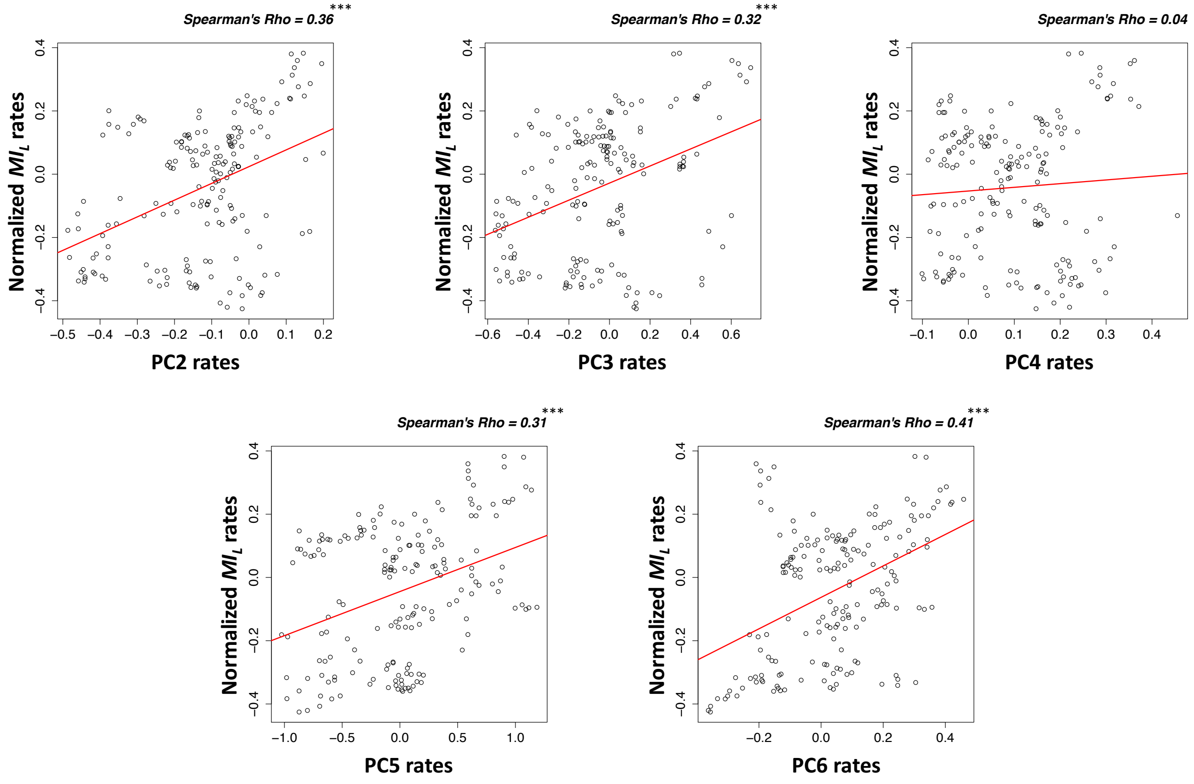

Supplementary Fig. 10. Correlations in evolutionary rates between acoustic structure (represented by PC2-6; see PC1 in the main text, Figure 4b) and local mutual information (MI_L). Rates were log transformed; significance levels: *P < 0.05; **P < 0.005; ***P < 0.001.

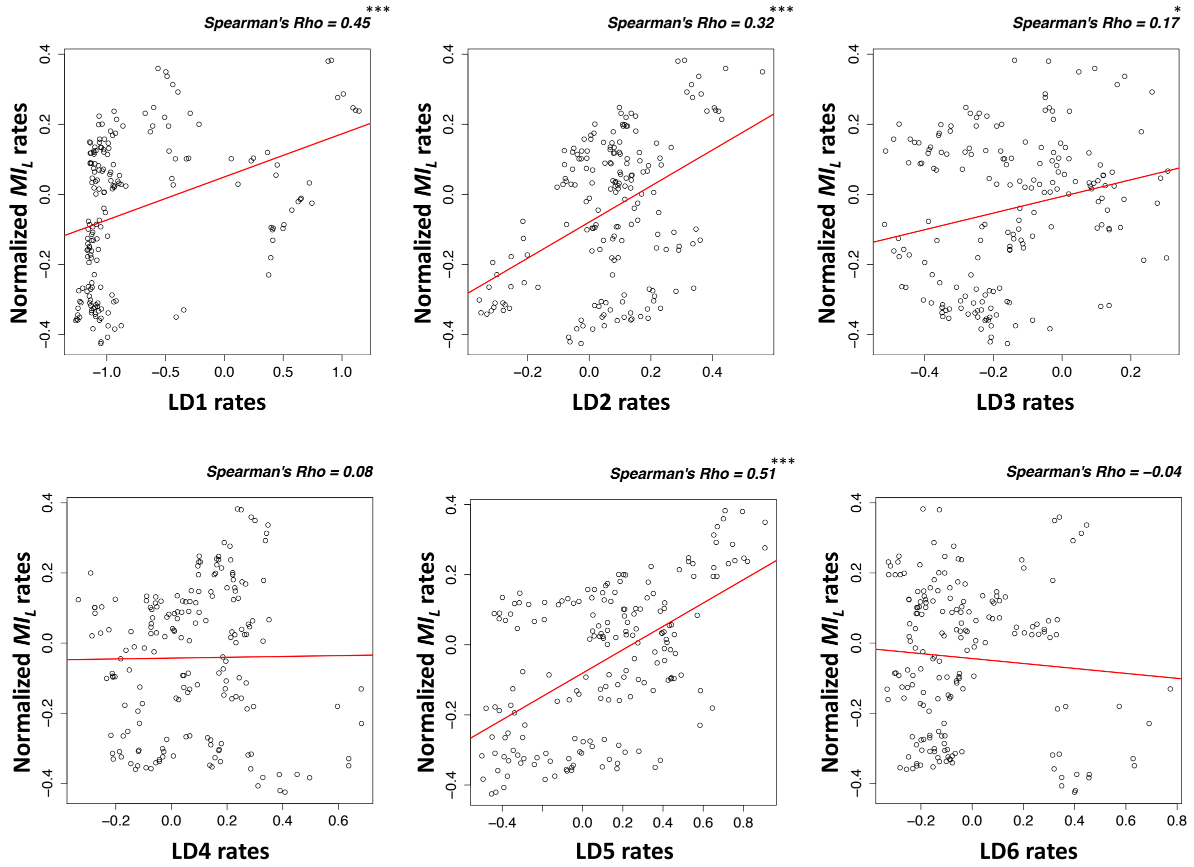

Supplementary Fig. 11. Similar to Supplementary Fig. 10, scatterplots highlighting the correlations in evolutionary rates between acoustic structure (represented by LD1-6 from left to right) and local mutual information (MI_L). Correlations show more variability than when using PCs. Rates were log transformed; significance levels: *P < 0.05; **P < 0.005; ***P < 0.001.

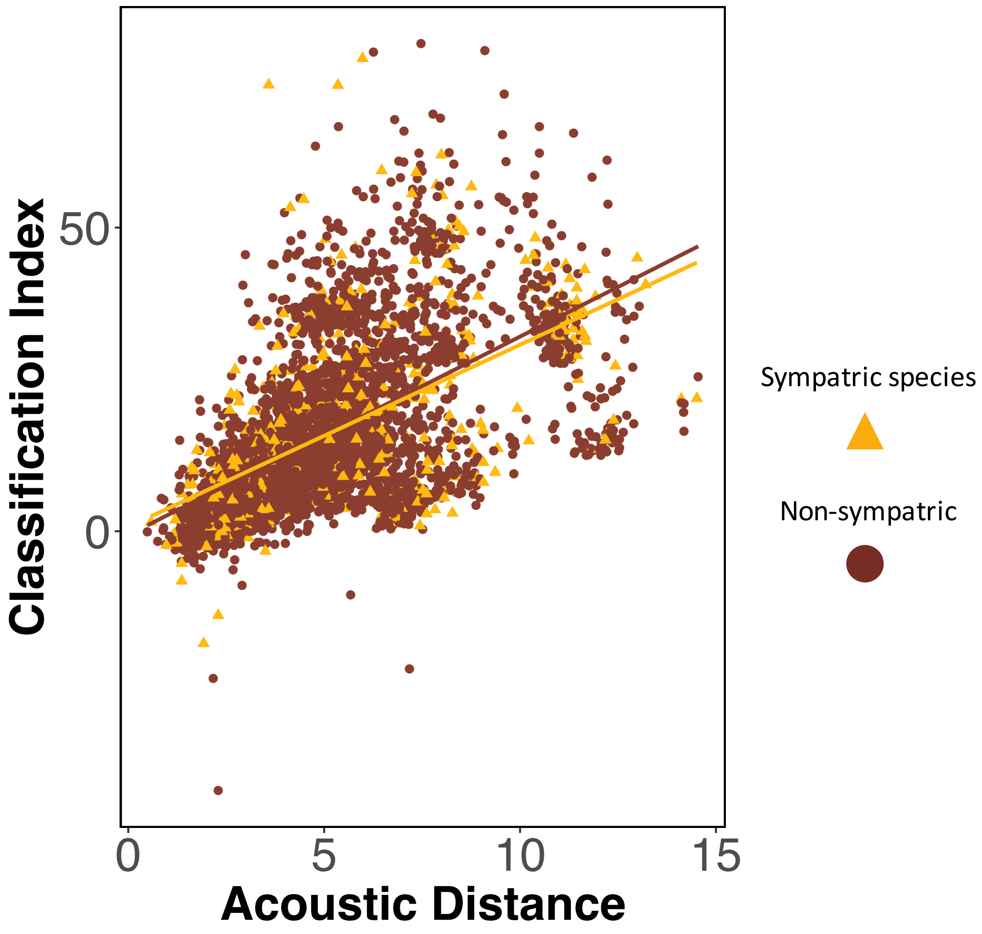

Supplementary Fig. 12. Relationship between acoustic distance and classification rate between species. Each dot represents a pair of species. Acoustic distances are computed as the Euclidean distance between 22-dimension vectors (for 22 drumming acoustic features; 1 vector per species). Classification index for an A-B pair is the average correct classification of species B and A (accounting for the misclassification of B in A and A in B – see methods for details). There is a significant positive effect of acoustic distance on classification, i.e. species pairs more distant acoustically are more likely to be correctly classified (Linear model: $\beta = 3.27$, $t = 42.64$, $P < 0.0001$), and sympatry has no effect on this relationship (Linear model: $\beta = 1.34$, $t = 1.29$, $P = 0.2$; Supplementary Table 10). For all linear models, two-tailed statistics are reported.

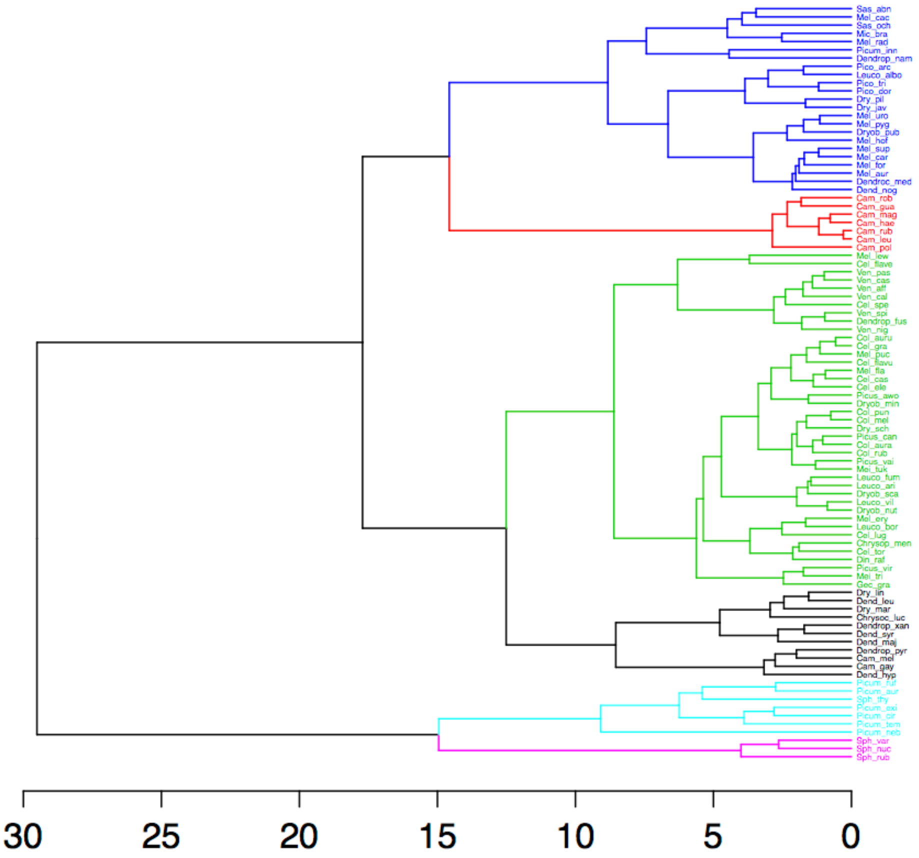

Supplementary Fig. 13. Dendrogram resulting from a hierarchical clustering using the 22 acoustic PCs instead of the 22 raw variables. Similar to Fig 2a, species can be classified into 6 main drumming clusters, which show very minor changes in species composition and branch lengths (as compared to Fig 2a).

RF Confusion Matrix 12.4 %

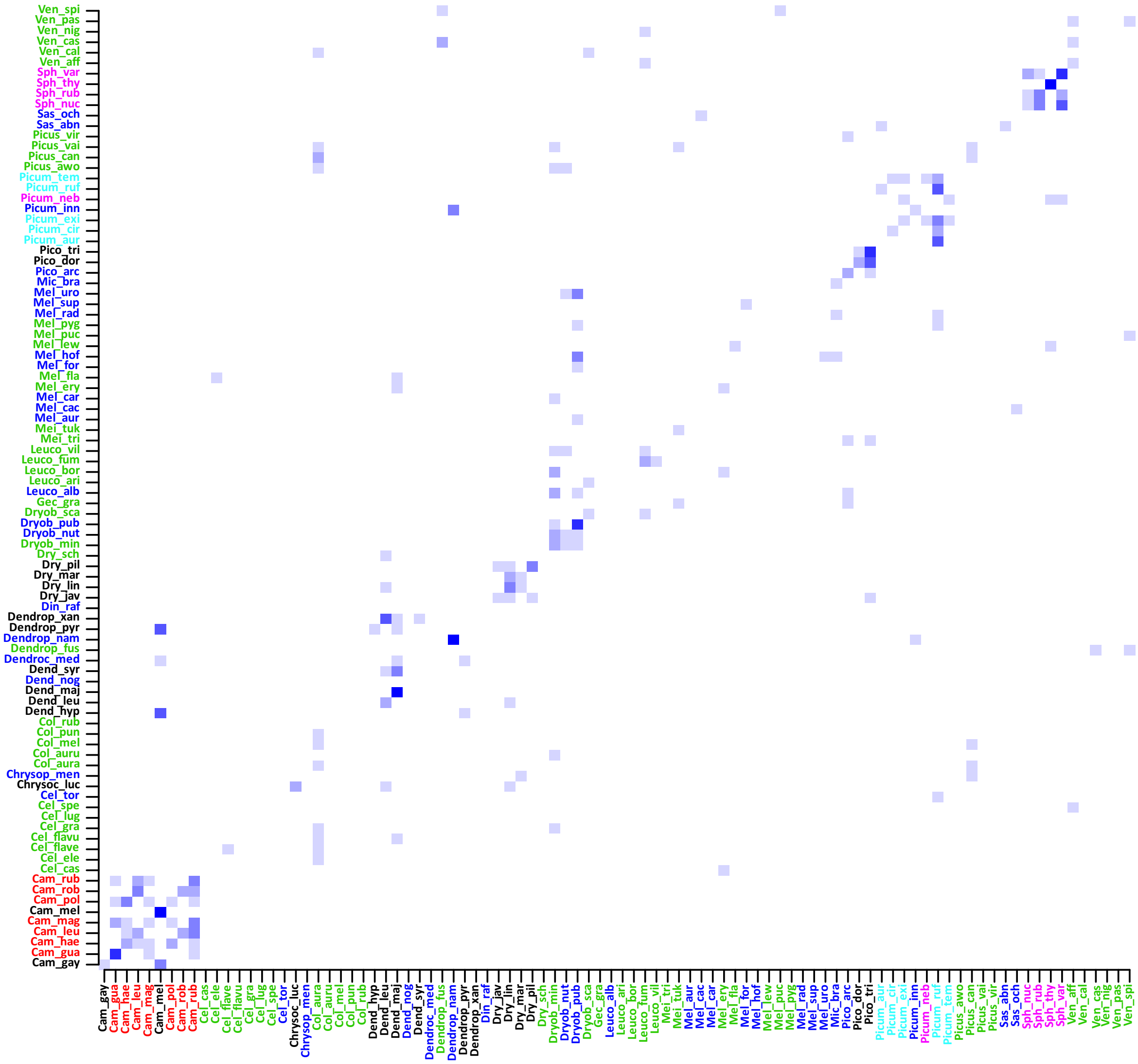

Supplementary Fig. 14. Confusion Matrix of the posterior probability of each drum to be correctly classified (i.e. to its own species) resulting from a Random Forest classification. The average rate of correct classification was 12.4 % versus 16.5% and the overall mutual information was 1.83 bits versus 2.50 bits relative to the DFA classification shown in Fig 2b. Note the localized increase in misclassification occurring among closely related species (illustrated by 'clusters' of blue squares: species are ordered alphabetically, thus species from a same genus are adjacent on the x and y axes). As in Fig. 2b, rows and columns correspond to actual and predicted species, respectively, and conditional probabilities are color-coded from light (low value) to dark (high value) blue.

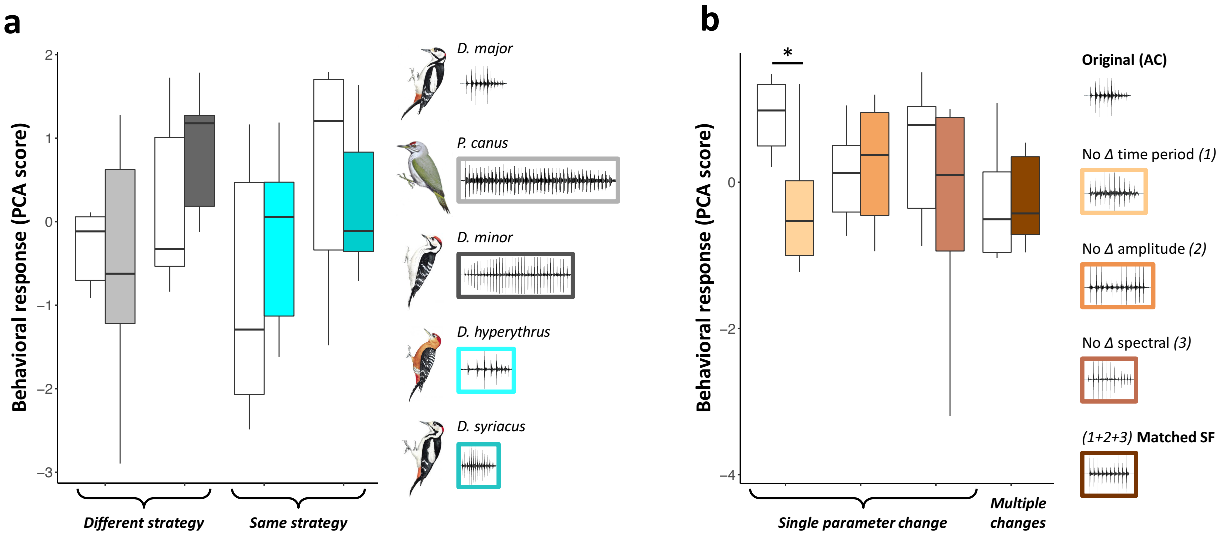

Supplementary Fig. 15. Behavioural response of great-spotted woodpecker *Dendrocopos major* to played back signals. As opposed to Fig. 5a-b where behavioural response is represented by the first principal component of the PCA made on behavioural data collected during playback experiments, here the behavioural response is represented as the second principal component of this PCA. No differences were seen **a)** between playback of conspecifics VS heterospecifics' drums, nor **b)** between conspecifics and resynthesized signals, besides a stronger drumming response to drums resynthesized without temporal variation than to *D.major* drums (β = - 0.61, t = - 2.15, P = 0.04). This overall indicates that drumming response of focal birds to our playback experiments did not differ across paired conditions (number of drums and latency to drum load onto the second principal component – see loadings in supplementary Table 13). For each of the experimental conditions, *n* = 6 individuals were tested twice (once with a conspecific drum, and once with either a heterospecific drum or a re-synthesized signal). In both panels, box plots denote median and 25th to 75th percentiles (boxes) and min/max values (whiskers). (Illustrations of woodpeckers reproduced by permission of Lynx Edicions). * significant difference between conditions using pairwise comparisons and fdr correction for multiple testing; two-tailed statistics are reported.

Variable	Description
n.pulses	number of pulses per drum
drum_duration	drum duration
pulseRateMedian	species specific median drum pulse rate
pulseRateDeviation	species specific median 'average pulse rate divided by median pulse rate' (represents the temporal jitter of a species specific drum)
interval_min	minimum time interval between 2 pulses in a drum
interval_max	maximum time interval between 2 pulses in a drum
interv1_intervMax	time interval between the first 2 pulses divided by 'interval_max' (indicates whether a drum starts quickly or slowly)
intervL_intervMax	time interval between the last 2 pulses divided by 'interval_max' (indicates whether a drum ends quickly or slowly)
interval_slope_lm2_ordre_coefa	first coefficient of the second order polynomial fit to consecutive inter-pulse intervals (proxy for the temporal dynamics/curve within a drum)
interval_nPVI	measure of temporal variation between consecutive inter-pulse intervals (similar to temporal jitter)
MeanAcc	mean acceleration coefficient of pulse rate within a drum
drum_peak_F	dominant frequency within a drum
Amp_pulses_min	relative minimum pulse amplitude within a drum
Amp_pulses_first	relative amplitude of the first pulse within a drum
Amp_pulses_last	relative amplitude of the last pulse within a drum
Amp_pulses_Q25	amplitude value below which 25% of pulse amplitude values are found within a drum
Amp_pulses_Q75	amplitude value below which 75% of pulse amplitude values are found within a drum
Amp_pulses_IQR	Amp_pulses_Q75' - 'median_Amp_pulses_Q25'
nPAVI	measure of amplitude variation between consecutive pulses (similar to amplitude jitter)
Amp_slope_lm2_ordre_coefa	first coefficient of the second order polynomial fit to consecutive pulse amplitude (proxy for the amplitude dynamics/curve within a drum)
n_seq	number of sequences of pulses within a drum
interval_seq_median	median time interval between sequences within a drum

Supplementary Table 1. Description of the 22 acoustic variables used to characterize drumming signals.

Reconstructed variable	Pagel's λ	Significance of phylogenetic signal	Ancestral state	CI 95% lower margin	CI 95% higher margin
**PC1**	0.925	P < 0.001	1.621	-1.488	4.73
**PC2**	0.823	P < 0.001	1.075	-1.64	3.79
**PC3**	0.893	P < 0.001	1.135	-0.858	3.129
**PC4**	0.905	P < 0.001	-0.758	-2.843	1.328
**PC5**	0.902	P < 0.001	-0.275	-1.781	1.23
**PC6**	0.899	P < 0.001	0.273	-0.832	1.378
**LD1**	0.941	P < 0.001	5.85	-3.909	15.609
**LD2**	0.911	P < 0.001	1.937	-3.092	6.967
**LD3**	0.905	P < 0.001	2.614	-1.368	6.595
**LD4**	0.792	P < 0.001	-0.075	-3.502	3.352
**LD5**	0.944	P < 0.001	-0.401	-2.14	1.339
**LD6**	0.679	P < 0.001	0.103	-2.575	2.781

Supplementary Table 2. Table summarizing the output of ancestral state reconstruction of PC1-PC6 and LD1-LD6. As PCs and LDs correspond to combinations of acoustic variables, their ancestral states and 95% confidence intervals have no unit.

PGLS on Local Mutual Information	Type of predictor variable	N	Intercept ± SE	Slope ± SE	λ	t (slope)	AICc	P
Null model (MI_L ~ 1)	Intercept only	92	0.57 ± 0.10	/	0.95	/	- 118.38	< 0.001
MI_L ~ Body Mass	Morphological	92	0.58 ± 0.10	0.02 ± 0.03	0.95	0.72	- 111.22	0.47
MI_L ~ Wing Length	Morphological	92	0.59 ± 0.10	0.03 ± 0.02	0.95	1.32	- 112.32	0.19
MI_L ~ Beak/Wing Ratio	Morphological	92	0.57 ± 0.10	0.01 ± 0.01	0.95	0.71	- 109.99	0.48
MI_L ~ Sympatry	Socio-Geographical	92	0.57 ± 0.10	0.01 ± 0.01	0.96	1.52	- 110.63	0.13
MI_L ~ Distribution Area	Socio-Geographical	92	0.57 ± 0.10	1 E-3 ± 0.01	0.94	- 0.08	- 108.88	0.94
**MI_L ~ PC1**	**Acoustic structure**	**92**	**0.51 ± 0.09**	**0.03 ± 0.01**	**0.94**	**4.06**	**- 123.69**	**< 0.001**
MI_L ~ PC2	Acoustic structure	92	0.55 ± 0.09	0.01 ± 0.01	0.93	1.20	- 110.07	0.23
MI_L ~ PC3	Acoustic structure	92	0.58 ± 0.10	- 0.01 ± 0.01	0.95	- 0.83	- 110.03	0.41
MI_L ~ PC4	Acoustic structure	92	0.55 ± 0.10	- 0.02 ± 0.01	0.94	- 1.92	- 112.88	0.06
MI_L ~ PC5	Acoustic structure	92	0.56 ± 0.10	- 0.02 ± 0.02	0.95	- 1.09	- 110.97	0.28
MI_L ~ PC6	Acoustic structure	92	0.57 ± 0.10	- 0.02 ± 0.02	0.95	- 0.83	- 111.19	0.41
**MI_L ~ LD1**	**Acoustic structure**	**92**	**0.49 ± 0.08**	**0.01 ± 3 E-3**	**0.89**	**5.08**	**- 127.07**	**< 0.001**
MI_L ~ LD2	Acoustic structure	92	0.56 ± 0.10	3 E-3 ± 5 E-3	0.94	0.54	- 107.81	0.59
MI_L ~ LD3	Acoustic structure	92	0.55 ± 0.10	7 E-3 ± 6 E-3	0.95	1.15	- 109.17	0.25
MI_L ~ LD4	Acoustic structure	92	0.57 ± 0.10	- 0.03 ± 8 E-3	0.97	- 3.70	- 119.98	< 0.001
MI_L ~ LD5	Acoustic structure	92	0.56 ± 0.10	- 0.01 ± 0.01	0.95	- 0.89	- 110.28	0.38
MI_L ~ LD6	Acoustic structure	92	0.57 ± 0.10	0.02 ± 0.01	0.95	1.57	- 111.27	0.12
MI_L ~ PC1 + PC2	Acoustic structure	92	0.47 ± 0.08	0.04 ± 8 E-3<br>0.03 ± 0.01	0.89	4.95<br>2.92	- 121.21	< 0.001 *(PC1)*<br>0.004 *(PC2)*
MI_L ~ PC1 + PC3	Acoustic structure	92	0.51 ± 0.09	0.03 ± 8 E-3<br>6 E-4 ± 0.01	0.94	3.95<br>0.05	- 114.51	< 0.001 *(PC1)*<br>0.96 *(PC3)*
MI_L ~ PC1 + PC4	Acoustic structure	92	0.49 ± 0.08	0.03 ± 8 E-3<br>- 0.03 ± 0.01	0.91	4.34<br>- 2 39	- 119.62	< 0.001 *(PC1)*<br>0.02 *(PC4)*
MI_L ~ PC1 + PC5	Acoustic structure	92	0.51 ± 0.09	0.03 ± 8 E-3<br>- 0.01 ± 0.02	0.94	4.01<br>- 0.97	- 115.83	< 0.001 *(PC1)*<br>0.34 *(PC5)*
MI_L ~ PC1 + PC6	Acoustic structure	92	0.52 ± 0.09	0.03 ± 8 E-3<br>- 0.01 ± 0.02	0.94	4.00<br>- 0.61	- 115.98	< 0.001 *(PC1)*<br>0.54 *(PC6)*
MI_L ~ LD1 + LD2	Acoustic structure	92	0.45 + 0.07	0.01 ± 3 E-3<br>0.01 ± 5 E-3	0.84	5.91<br>2.37	-120.95	< 0.001 *(LD1)*<br>0.02 *(LD2)*
MI_L ~ LD1 + LD3	Acoustic structure	92	0.48 ± 0.08	0.01 ± 3 E-3<br>3 E-3 ± 6 E-3	0.90	4.92<br>0.58	- 116.63	< 0.001 *(LD1)*<br>0.56 *(LD3)*
MI_L ~ LD1 + LD4	Acoustic structure	92	0.50 ± 0.08	0.01 ± 3 E-3<br>- 0.01 ± 8 E-3	0.91	3.97<br>- 1.81	- 120.15	< 0.001 *(LD1)*<br>0.07 *(LD4)*
MI_L ~ LD1 + LD5	Acoustic structure	92	0.49 ± 0.08	0.01 ± 3 E-3<br>- 3 E-3 ± 0.01	0.89	4.93<br>- 0.24	- 117.98	< 0.001 *(LD1)*<br>0.81 *(LD5)*
MI_L ~ LD1 + LD6	Acoustic structure	92	0.49 ± 0.08	0.01 ± 3 E-3<br>5 E-3 ± 1 E-3	0.89	4.83<br>0.53	- 117.74	< 0.001 *(LD1)*<br>0.59 *(LD6)*

In two cases, a single variable addition significantly improved the model (decrease in AICc >2): PC1 and LD1.
Adding more variables (i.e. models with 2 predictive variables) did not significantly further improve the models' fit.

Supplementary Table 3. Output from PGLS models testing for an effect of life history traits and acoustic structure on Local Mutual Information (MI_L). Model selection following a stepwise forward approach. Models with a significant decrease in AICc (> 2) for an added variable are in bold. Two-tailed statistics are reported.

PGLS PC6 VS Body Mass	Type of predictor variable	N	Intercept ± SE	Slope ± SE	λ	t (slope)	AICc	P
Null model (PC6 ~ 1)	Intercept only	92	0.27 ± 0.41	/	0.91	/	155.74	0.51
PC6 ~ Body Mass	Morphological	92	***0.09 ± 0.35	- 0.36 ± 0.10	0.85	- 3.56	**149.39**	**< 0.001**
**LRT BodyMass-fitted model / NULL model**	**DF = 1**		**LR = 11.63**	**P < 0.001**				

PGLS PC6 VS Wing Length	Type of predictor variable	N	Intercept ± SE	Slope ± SE	λ	t (slope)	AICc	P
Null model (PC6 ~ 1)	Intercept only	92	0.27 ± 0.41	/	0.91	/	155.74	0.51
PC6 ~ Wing Length	Morphological	92	**0.02 ± 0.36	- 0.29 ± 0.10	0.84	- 2.89	**153.33**	**0.005**
**LRT BodyMass-fitted model / NULL model**	**DF = 1**		**LR = 7.63**	**P = 0.006**				

PGLS MI_L VS PC1	Type of predictor variable	N	Intercept ± SE	Slope ± SE	λ	t (slope)	AICc	P
Null model (MI_L ~ 1)	Intercept only	92	***0.57 ± 0.10	/	0.95	/	- 118.38	< 0.001
MI_L ~ PC1	Acoustic structure	92	***0.51 ± 0.09	0.03 ± 0.01	0.94	4.06	- 123.69	< 0.001
**LRT PC1-fitted model / NULL model**	**DF = 1**		**LR = 15.48**	**P < 0.001**				

PGLS MIL VS LD1	Type of predictor variable	N	Intercept ± SE	Slope ± SE	λ	t (slope)	AICc	P
Null model (MI_L ~ 1)	Intercept only	92	***0.57 ± 0.10	/	0.95	/	- 118.38	< 0.001
MI_L ~ LD1	Acoustic structure	92	***0.49 ± 0.08	0.01 ± 3 E-3	0.89	5.08	- 127.07	< 0.001
**LRT LD1-fitted model / NULL model**	**DF = 1**		**LR = 21.45**	**P < 0.001**				

Supplementary Table 4. Summary output of the Likelihood Ratio Tests conducted between full and NULL models testing the effect of predictive variables significantly lowering the models' fits, respectively in the PGLS on acoustic structure (two top tables) and on Local Mutual Information (MI_L) (two bottom tables). Two-tailed statistics are reported.

PGLS on acoustic structure (PC1)	Type of predictor variable	N	Intercept ± SE	Slope ± SE	λ	t (slope)	AICc	P
Null model (PC1 ~ 1)	Intercept only	92	1.6 ± 1.12	/	0.93	/	331.68	0.16
PC1 ~ Body Mass	Morphological	92	1.58 ± 1.13	- 0.05 ± 0.30	0.93	- 0.16	334.41	0.87
PC1 ~ Wing Length	Morphological	92	1.58 ± 1.15	- 0.02 ± 0.29	0.93	- 0.07	334.5	0.95
PC1 ~ Beak/Wing Ratio	Morphological	92	1.6 ± 1.12	- 0.05 ± 0.17	0.93	- 0.30	335.51	0.76
PC1 ~ Sympatry	Socio-Geographical	92	1.63 ± 1.13	0.20 ± 0.12	0.94	1.65	333.67	0.10
PC1 ~ Distribution Area	Socio-Geographical	92	1.62 ± 1.13	0.08 ± 0.12	0.93	0.64	335.78	0.52

No variable addition significantly improved the model (no decrease in AICc >2).

PGLS on acoustic structure (PC2)	Type of predictor variable	N	Intercept ± SE	Slope ± SE	λ	t (slope)	AICc	P
Null model (PC2 ~ 1)	Intercept only	92	1.07 ± 0.77	/	0.83	/	295.77	0.17
PC2 ~ Body Mass	Morphological	92	1.07 ± 0.78	8 E-3 ± 0.23	0.83	0.04	299.06	0.97
PC2 ~ Wing Length	Morphological	92	1.07 ± 0.80	5 E-3 ± 0.23	0.83	0.02	299.09	0.98
PC2 ~ Beak/Wing Ratio	Morphological	92	1.07 ± 0.77	- 0.13 ± 0.14	0.83	- 0.96	299.14	0.34
PC2 ~ Sympatry	Socio-Geographical	92	1.05 ± 0.80	- 0.22 0.11	0.86	-2.03	296.71	0.04
PC2 ~ Distribution Area	Socio-Geographical	92	1.04 ± 0.77	- 0.12 ± 0.11	0.84	- 1.11	299.31	0.27

No variable addition significantly improved the model (no decrease in AICc >2).

PGLS on acoustic structure (PC3)	Type of predictor variable	N	Intercept ± SE	Slope ± SE	λ	t (slope)	AICc	P
Null model (PC3 ~ 1)	Intercept only	92	1.09 ± 0.72	/	0.90	/	260.5	0.13
PC3 ~ Body Mass	Morphological	92	1.04 ± 0.72	- 0.11 ± 0.20	0.90	- 0.58	263.76	0.57
PC3 ~ Wing Length	Morphological	92	0.95 ± 0.72	- 0.16 ± 0.19	0.89	- 0.83	263.48	0.41
PC3 ~ Beak/Wing Ratio	Morphological	92	1.09 ± 0.72	0.10 ± 0.11	0.91	0.88	264.43	0.38
PC3 ~ Sympatry	Socio-Geographical	92	1.09 ± 0.77	- 0.15 ± 0.08	0.94	- 1.94	262.65	0.06
PC3 ~ Distribution Area	Socio-Geographical	92	1.09 ± 0.72	- 0.03 ± 0.09	0.90	- 0.34	265.62	0.74

No variable addition significantly improved the model (no decrease in AICc >2).

PGLS on acoustic structure (PC4)	Type of predictor variable	N	Intercept ± SE	Slope ± SE	λ	t (slope)	AICc	P
Null model (PC4 ~ 1)	Intercept only	92	- 0.72 ± 0.76	/	0.91	/	267.86	0.34
PC4 ~ Body Mass	Morphological	92	- 0.88 ± 0.76	- 0.32 ± 0.21	0.91	- 1.55	268.96	0.12
PC4 ~ Wing Length	Morphological	92	- 0.96 ± 0.78	- 0.28 ± 0.20	0.91	- 1.39	269.48	0.17
PC4 ~ Beak/Wing Ratio	Morphological	92	- 0.72 ± 0.76	- 0.02 ± 0.12	0.91	- 0.16	272.45	0.88
PC4 ~ Sympatry	Socio-Geographical	92	- 0.74 ± 0.78	- 0.06 ± 0.09	0.92	- 0.72	272.67	0.48
PC4 ~ Distribution Area	Socio-Geographical	92	- 0.75 ± 0.76	- 0.10 ± 0.09	0.91	- 1.10	271.83	0.27

No variable addition significantly improved the model (no decrease in AICc >2).

PGLS on acoustic structure (PC5)	Type of predictor variable	N	Intercept ± SE	Slope ± SE	λ	t (slope)	AICc	P
Null model (PC5 ~ 1)	Intercept only	92	- 0.30 ± 0.59	/	0.91	/	219.85	0.61
PC5 ~ Body Mass	Morphological	92	- 0.29 ± 0.6	0.01 ± 0.16	0.92	0.09	223.84	0.93
PC5 ~ Wing Length	Morphological	92	- 0.28 ± 0.61	0.01 ± 0.16	0.92	0.09	223.9	0.93
PC5 ~ Beak/Wing Ratio	Morphological	92	- 0.29 ± 0.61	- 0.11 ± 0.09	0.94	- 1.21	223.69	0.23
PC5 ~ Sympatry	Socio-Geographical	92	- 0.30 ± 0.58	0.02 ± 0.07	0.91	0.28	225.49	0.78
PC5 ~ Distribution Area	Socio-Geographical	92	- 0.29 ± 0.57	0.05 ± 0.07	0.90	0.76	224.97	0.45

No variable addition significantly improved the model (no decrease in AICc >2).

PGLS on acoustic structure (PC6)	Type of predictor variable	N	Intercept ± SE	Slope ± SE	λ	t (slope)	AICc	P	
Null model (PC6 ~ 1)	Intercept only	92	0.27 ± 0.41	/	0.91	/	155.74	0.51	
**PC6 ~ Body Mass**	**Morphological**	**92**	**0.09 ± 0.35**	**- 0.36 ± 0.10**	**0.85**	**- 3.56**	**149.39**	**< 0.001**	
**PC6 ~ Wing Length**	**Morphological**	**92**	**0.02 ± 0.36**	**- 0.29 ± 0.10**	**0.84**	**- 2.89**	**153.33**	**0.005**	
PC6 ~ Beak/Wing Ratio	Morphological	92	0.27 ± 0.39	- 0.08 ± 0.06	0.88	- 1.31	159.99	0.19	
PC6 ~ Sympatry	Socio-Geographical	92	0.27 ± 0.42	- 0.04 ± 0.05	0.92	- 0.86	161.49	0.39	
PC6 ~ Distribution Area	Socio-Geographical	92	0.25 ± 0.41	- 0.08 ± 0.05	0.91	- 1.64	159.5	0.10	
PC6 ~ Body Mass + Wing Length	Morphological	92	0.12 ± 0.36	- 0.42 ± 0.21	0.85	- 2.01	152.87	0.05	*(Body Mass)*
				0.06 ± 0.20		0.31		0.76	*(Wing Length)*
PC6 ~ Body Mass + Beak/Wing Ratio	Morphological	92	0.10 ± 0.35	- 0.035 ± 0.11	0.84	- 3.26	155.08	0.002	*(Body Mass)*
				- 0.03 ± 0.06		- 0.47		0.64	*(Beak/Wing Ratio)*
PC6 ~ Body Mass + Sympatry	Combined	92	0.09 ± 0.36	- 0.037 ± 0.10	0.86	- 3.58	154.92	< 0.001	*(Body Mass)*
				- 0.05 ± 0.05		- 0.98		0.33	*(Sympatry)*
PC6 ~ Body Mass + Distribution Area	Combined	92	0.09 ± 0.35	- 0.34 ± 0.10	0.85	- 3.35	154.49	0.001	*(Body Mass)*
				- 0.06 ± 0.05		- 1.17		0.25	*(Distribution Area)*
PC6 ~ Wing Length + Beak/Wing Ratio	Morphological	92	0.034 ± 0.35	- 0.28 ± 0.10	0.83	- 2.75	158.14	0.007	*(Wing Length)*
				- 0.07 ± 0.06		- 1.07		0.29	*(Beak/Wing Ratio)*
PC6 ~ Wing Length + Sympatry	Combined	92	0.02 ± 0.37	- 0.30 ± 0.10	0.85	- 2.88	159.24	0.005	*(Wing Length)*
				- 0.04 ± 0.05		- 0.71		0.48	*(Sympatry)*
PC6 ~ Wing Length + Distribution Area	Combined	92	0.02 ± 0.36	- 0.27 ± 0.10	0.85	- 2.66	158.33	0.01	*(Wing Length)*
				- 0.06 ± 0.05		- 1.19		0.24	*(Distribution Area)*

In two cases, a single variable addition significantly improved the model (decrease in AICc >2): Body Mass and Wing Length.
Adding more variables (i.e. models with 2 predictive variables) did not significantly further improve the models' fit.

Supplementary Table 5. Output from PGLS models testing for an effect of life history traits on acoustic structure (PC1-PC6). Model selection following a stepwise forward approach. Models with a significant decrease in AICc (> 2) for an added variable are in bold. Two-tailed statistics are reported.

PGLS on acoustic structure (LD1)	Type of predictor variable	N	Intercept ± SE	Slope ± SE	λ	t (slope)	AICc	P
Null model (LD1 ~ 1)	Intercept only	92	5.82 ± 3.52	/	0.94	/	534.29	0.10
LD1 ~ Body Mass	Morphological	92	5.69 ± 3.56	- 0.27 ± 0.93	0.94	- 0.29	534.71	0.11
LD1 ~ Wing Length	Morphological	92	5.67 ± 3.62	- 0.18 ± 0.89	0.94	- 0.20	534.83	0.84
LD1 ~ Beak/Wing Ratio	Morphological	92	5.85 ± 3.52	- 0.63 ± 0.50	0.94	- 1.25	534.45	0.21
LD1 ~ Sympatry	Socio-Geographical	92	5.85 ± 3.52	0.18 ± 0.36	0.94	0.49	536.44	0.62
LD1 ~ Distribution Area	Socio-Geographical	92	5.81 ± 3.53	- 0.06 ± 0.37	0.94	- 0.16	536.59	0.88

No variable addition significantly improved the model (no decrease in AICc >2).

PGLS on acoustic structure (LD2)	Type of predictor variable	N	Intercept ± SE	Slope ± SE	λ	t (slope)	AICc	P
Null model (LD2 ~ 1)	Intercept only	92	1.88 ± 1.83	/	0.92	/	425.95	0.31
LD2 ~ Body Mass	Morphological	92	2.13 ± 1.84	0.52 ± 0.50	0.91	1.05	426.60	0.30
LD2 ~ Wing Length	Morphological	92	2.29 ± 1.88	0.48 ± 0.48	0.92	1.00	426.76	0.32
LD2 ~ Beak/Wing Ratio	Morphological	92	1.88 ± 1.85	- 0.05 ± 0.28	0.92	- 0.19	428.80	0.85
LD2 ~ Sympatry	Socio-Geographical	92	1.83 ± 1.80	- 0.32 ± 0.21	0.91	- 1.51	427.17	0.13
LD2 ~ Distribution Area	Socio-Geographical	92	1.87 ± 1.83	- 0.03 ± 0.21	0.91	- 0.12	429.38	0.91

No variable addition significantly improved the model (no decrease in AICc >2).

PGLS on acoustic structure (LD3)	Type of predictor variable	N	Intercept ± SE	Slope ± SE	λ	t (slope)	AICc	P
Null model (LD3 ~ 1)	Intercept only	92	2.51 ± 1.52	/	0.91	/	394.54	0.10
LD3 ~ Body Mass	Morphological	92	2.31 ± 1.50	- 0.40 ± 0.41	0.90	- 0.97	395.75	0.33
LD3 ~ Wing Length	Morphological	92	2.07 ± 1.52	- 0.50 ± 0.40	0.90	- 1.25	395.21	0.21
LD3 ~ Beak/Wing Ratio	Morphological	92	2.51 ± 1.55	0.24 ± 0.24	0.92	1.00	396.79	0.32
LD3 ~ Sympatry	Socio-Geographical	92	2.52 ± 1.52	0.07 ± 0.18	0.91	0.40	398.18	0.69
LD3 ~ Distribution Area	Socio-Geographical	92	2.53 ± 1.53	0.06 ± 0.18	0.91	0.34	398.20	0.73

No variable addition significantly improved the model (no decrease in AICc >2).

PGLS on acoustic structure (LD4)	Type of predictor variable	N	Intercept ± SE	Slope ± SE	λ	t (slope)	AICc	P
Null model (LD4 ~ 1)	Intercept only	92	- 0.09 ± 0.93	/	0.80	/	337.35	0.93
LD4 ~ Body Mass	Morphological	92	- 0.32 ± 0.94	- 0.50 ± 0.28	0.81	- 1.79	337.06	0.08
LD4 ~ Wing Length	Morphological	92	- 0.49 ± 0.93	- 0.50 ± 0.28	0.80	- 1.81	337.03	0.07
LD4 ~ Beak/Wing Ratio	Morphological	92	- 0.08 ± 092	- 0.18 ± 0.17	0.80	- 1.05	340.08	0.29
LD4 ~ Sympatry	Socio-Geographical	92	- 0.11 ± 0.97	- 0.22 ± 0.14	0.84	- 1.56	339.40	0.12
LD4 ~ Distribution Area	Socio-Geographical	92	- 0.13 ± 0.94	- 0.17 ± 0.14	0.81	- 1.25	340.09	0.21

No variable addition significantly improved the model (no decrease in AICc >2).

PGLS on acoustic structure (LD5)	Type of predictor variable	N	Intercept ± SE	Slope ± SE	λ	t (slope)	AICc	P
Null model (LD5 ~ 1)	Intercept only	92	- 0.42 ± 0.74	/	0.95	/	248.56	0.58
LD5 ~ Body Mass	Morphological	92	- 0.33 ± 0.74	0.18 ± 0.19	0.95	0.91	251.38	0.37
LD5 ~ Wing Length	Morphological	92	- 0.27 ± 0.76	0.18 ± 0.19	0.95	0.95	251.37	0.34
LD5 ~ Beak/Wing Ratio	Morphological	92	- 0.41 ± 0.78	- 0.09 ± 0.10	0.97	- 0.90	252.83	0.37
LD5 ~ Sympatry	Socio-Geographical	92	- 0.42 ± 0.74	0.01 ± 0.07	0.94	0.18	254.09	0.86
LD5 ~ Distribution Area	Socio-Geographical	92	- 0.40 ± 0.72	0.09 ± 0.08	0.94	1.17	252.71	0.25

No variable addition significantly improved the model (no decrease in AICc >2).

PGLS on acoustic structure (LD6)	Type of predictor variable	N	Intercept ± SE	Slope ± SE	λ	t (slope)	AICc	P
Null model (LD6 ~ 1)	Intercept only	92	0.06 ± 062	/	0.70	/	290.33	0.93
LD6 ~ Body Mass	Morphological	92	0.03 ± 0.63	- 0.07 ± 0.21	0.70	- 0.34	293.71	0.73
LD6 ~ Wing Length	Morphological	92	0.06 ± 0.65	4.9 E-3 ± 0.21	0.71	0.02	293.84	0.98
LD6 ~ Beak/Wing Ratio	Morphological	92	0.06 ± 0.62	0.03 ± 0.13	0.70	0.24	294.63	0.81
LD6 ~ Sympatry	Socio-Geographical	92	0.08 ± 0.67	0.14 ± 0.11	0.75	1.21	293.80	0.23
LD6 ~ Distribution Area	Socio-Geographical	92	0.07 ± 0.62	0.06 ± 0.11	0.70	0.53	294.75	0.60

No variable addition significantly improved the model (no decrease in AICc >2).

Supplementary Table 6. Output from PGLS models testing for an effect of life history traits on acoustic structure (LD1-LD6). Model selection following a stepwise forward approach. No variable improved significantly the NULL model's fit for any of the trait (LD1-LD6) considered. Two-tailed statistics are reported.

Variable pair	Spearman Rho	P *(Spearman)*	Wilcoxon's Z	P *(Wilcoxon)*
PC1 vs. MI_L	0.13	0.08	-8.08	< 0.001
PC2 vs. MI_L	0.36	< 0.001	-5.25	< 0.001
PC3 vs. MI_L	0.32	< 0.001	-1.00	1
PC4 vs. MI_L	0.04	0.59	-6.61	< 0.001
PC5 vs. MI_L	0.31	< 0.001	-1.65	< 0.001
PC6 vs. MI_L	0.41	< 0.001	-5.63	< 0.001
LD1 vs. MI_L	0.45	< 0.001	-10.51	< 0.001
LD2 vs. MI_L	0.32	< 0.001	-5.79	< 0.001
LD3 vs. MI_L	0.17	0.02	-4.95	< 0.001
LD4 vs. MI_L	0.08	0.3	-4.83	< 0.001
LD5 vs. MI_L	0.51	< 0.001	-6.53	< 0.001
LD6 vs. MI_L	-0.04	0.62	-0.82	1

Supplementary Table 7. Summary output of the comparisons carried out using evolutionary rates (log transformed) between drumming acoustic structure (PC1-PC6 and LD1-LD6) and local mutual information (MI_L). Correlations were conducted using Spearman's rank correlation coefficients; Bonferroni correction for multiple testing was applied to P values resulting from pairwise Wilcoxon signed rank tests and two-tailed statistics are reported.

Community	Switzerland (Collinean and mountain zone)	Drummer status	Guatemala (Tikal National Park)	Drummer status
Species	***Dendrocopos major*** **(Great spotted woodpecker)**	**Y**	***Campephilus guatemalensis*** **(Pale-billed woodpecker)**	**Y**
	Dendrocoptes medius **(Middle spotted woodpecker)**	**Y**	***Celeus castaneus*** **(Chestnut-colored woodpecker)**	**Y**
	Dryobates minor **(Lesser spotted woodpecker)**	**Y**	***Colaptes rubiginosus*** **(Golden-olive woodpecker)**	**Y**
	Dryocopus martius **(Black woodpecker)**	**Y**	***Dryocopus lineatus*** **(Lineated woodpecker)**	**Y**
	Picus Canus **(Grey-headed woodpecker)**	**Y**	***Leuconotopicos fumigatus*** **(Smoky-brown woodpecker)**	**Y**
	Picus viridis **(Eurasian green woodpecker)**	**Y**	***Melanerpes aurifrons*** **(Golden-fronted woodpecker)**	**Y**
			Melanerpes pucherani **(Black-cheeked woodpecker)**	**Y**
Source	[1]	[2]	[3]	[2]

Community	Minnesota (Chain of Lakes Park)	Drummer status	Malaysia (Sungai Lalang Forest Reserve)	Drummer status
Species	***Colaptes auratus*** **(Common flicker)**	**Y**	*Blythipicus rubiginosus* (Maroon woodpecker)	N
	Dryobates pubescens **(Downy woodpecker)**	**Y**	***Chrysophlegma mentale*** **(Chequer-throated woodpecker)**	**Y**
	Dryocopus pileatus **(Pileated woodpecker)**	**Y**	*Chrysophlegma miniaceum* (Banded woodpecker)	N
	Leuconotopicos villosus **(Hairy woodpecker)**	**Y**	***Dinopium rafflesii*** **(Olive-backed woodpecker)**	**Y**
	Melanerpes carolinus **(Red-bellied woodpecker)**	**Y**	***Dryocopus javensis*** **(White-bellied woodpecker)**	**Y**
	Melanerpes erythrocephalus **(Red-headed woodpecker)**	**Y**	*Hemicircus concretus* (Grey-and-buff woodpecker)	N
	Sphyrapicus varius **(Yellow-bellied sapsucker)**	**Y**	***Meiglyptes tristis*** **(Buff-rumped woodpecker)**	**Y**
			Meiglyptes tukki **(Buff-necked woodpecker)**	**Y**
			Micropternus brachyurus **(Rufous woodpecker)**	**Y**
			Mulleripicus pulverulentus (Great slaty woodpecker)	N
			Picus puniceus (Crimson-winged woodpecker)	Y
			Reinwardtipicus validus (Orange-backed woodpecker)	Y
			Sasia abnormis **(Rufous piculet)**	**Y**
Source	[3]	[2]	[4]	[2]

Community	French Guiana (Réserve des Nouragues)	Drummer status
Species	***Campephilus melanoleucos*** **(Crimson-crested woodpecker)**	**Y**
	Campephilus rubricollis **(Red-necked woodpecker)**	**Y**
	Celeus elegans **(Chestnut woodpecker)**	**Y**
	Celeus torquatus **(Ringed woodpecker)**	**Y**
	Celeus undatus (Waved woodpecker)	Y
	Colaptes rubiginosus **(Golden-olive woodpecker)**	**Y**
	Dryocopus lineatus **(Lineated woodpecker)**	**Y**
	Melanerpes cruentatus (Yellow-tufted woodpecker)	N
	Piculus chrysochloros (Golden-green woodpecker)	Y
	Piculus flavigula (Yellow-throated woodpecker)	Y
	Picumnus exilis **(Golden-spangled piculet)**	**Y**
	Veniliornis cassini **(Golden-collared woodpecker)**	**Y**
Source	[5]	[2]

Supplementary Table 8. Species composition of the communities used in the ecological investigation of drumming types distribution. Y: Species with a documented drumming behavior. N: Species either documented as non-drumming, or without proper drumming documented. Species in bold are those for which drumming has been documented and that are included in our community analysis (i.e. for which drumming data could be collected).

	Comparison	Wilcoxon's Z	P *(Wilcoxon)*
**Acoustic Distance**	Global vs. Switzerland	-3.51	**0.002**
	Global vs. Guatemala	-2.77	**0.03**
	Global vs. Minnesota	-0.17	1
	Global vs. Malaysia	-2.14	0.16
	Global vs. French Guiana	-0.10	1
**Classification Index**	Global vs. Switzerland	-5.95	**< 0.001**
	Global vs. Guatemala	-6.12	**< 0.001**
	Global vs. Minnesota	-7.56	**< 0.001**
	Global vs. Malaysia	-3.28	**0.005**
	Global vs. French Guiana	-7.09	**< 0.001**

Supplementary Table 9. Summary output of the comparisons between acoustic distances found in the woodpecker family as a whole ('Global') vs. those found within communities, and between the classification indexes found taking the woodpecker family as a whole vs. those found within communities. Bonferroni correction for multiple testing was applied to P values resulting from pairwise Wilcoxon Rank Sum tests. Significant differences are in bold and two-tailed statistics are reported.

While the majority of acoustic distance distributions are found to be similar in communities and in the family as a whole (only in the Swiss community we found a clearly significant difference, Guatemala being only moderately significant), all comparisons showed highly significant differences between the classification distributions found in the entire family and in communities.

Model: AD ~ PD + S + PD*S (corresponds to Fig. 7a)	Estimate ± SE	t	P
PD	5.92 ± 0.21	28.31	< 0.001
S	2.93 ± 0.57	5.11	< 0.001
PD*S	- 1.8 ± 0.41	- 4.41	< 0.001

AD: Acoustic Distance; PD: Phylogenetic Distance; S: Sympatry

Model: CI ~ PD + S + PD*S (corresponds to Fig. 7b)	Estimate ± SE	t	P
PD	15.95 ± 1.26	12.71	< 0.001
S	15.51 ± 3.44	4.51	< 0.001
PD*S	-10.33 ± 2.44	- 4.23	< 0.001

CI: Classification Index; PD: Phylogenetic Distance; S: Sympatry

Model: CI ~ AD + S + AD*S (corresponds to Sup. Fig. 12)	Estimate ± SE	t	P
AD	3.27 ± 0.08	42.64	< 0.001
S	1.34 ± 1.04	1.29	0.196
AD*S	- 0.27 ± 0.18	- 1.48	0.138

CI: Classification Index; AD: Acoustic Distance; S: Sympatry

Supplementary Table 10. Summary output from Linear models testing for the relationship between acoustic distance, phylogenetic distance, classification index and sympatry. For all Linear models, two-tailed statistics are reported.

	PC1	PC2	PC3	PC4	PC5	PC6
**n.pulses**	0.57	-0.36	0.55	0.09	0.13	-0.17
**drum_duration**	0.70	0.10	0.51	-0.18	0.20	-0.18
**pulseRateMedian**	0.10	-0.56	-0.01	0.71	-0.24	0.11
**pulseRateDeviation**	-0.17	-0.10	-0.05	-0.22	0.72	-0.19
**interval_min**	-0.39	0.54	0.16	-0.57	0.08	-0.05
**interval_max**	0.69	0.55	0.16	-0.19	-0.31	-0.07
**interv1_intervMax**	-0.69	-0.46	-0.30	0.01	0.16	-0.02
**intervL_intervMax**	-0.72	-0.21	0.28	0.17	0.27	0.05
**interval_slope_lm2_ordre_coefa**	-0.10	0.07	-0.39	-0.20	-0.36	0.21
**interval_nPVI**	0.81	0.46	-0.23	0.20	0.05	0.07
**MeanAcc**	0.70	0.35	-0.44	0.27	0.10	0.08
**drum_peak_F**	0.22	-0.04	0.27	-0.02	0.06	0.71
**Amp_pulses_min**	-0.60	0.55	-0.03	0.41	-0.02	-0.11
**Amp_pulses_first**	-0.13	0.23	-0.52	0.40	-0.10	-0.45
**Amp_pulses_last**	-0.40	0.53	0.40	0.31	-0.05	0.25
**Amp_pulses_Q25**	-0.65	0.53	0.28	0.24	0.10	0.01
**Amp_pulses_Q75**	-0.45	0.48	-0.22	-0.08	0.25	0.13
**Amp_pulses_IQR**	0.49	-0.31	-0.52	-0.36	0.05	0.09
**nPAVI**	0.85	-0.07	0.08	0.15	0.18	-0.02
**Amp_slope_lm2_ordre_coefa**	0.20	-0.18	0.40	0.32	0.06	-0.10
**n_seq**	0.77	0.43	-0.04	0.17	0.25	-0.03
**interval_seq_median**	-0.11	-0.02	0.38	-0.27	-0.64	-0.26
**Explained variance**	29.37%	14.18%	10.95%	9.06%	7.15%	4.81%
**Cumulative explained variance**	**29.37%**	**43.56%**	**54.51%**	**63.57%**	**70.72%**	**75.53%**

Supplementary Table 11. Loading scores of the 22 acoustic variables on the first 6 principal components (PCs) resulting from the Principal Component Analysis carried out on these variables. Individual and cumulative variance explained by these PCs are indicated in the lower lines of the table.

	LD1	LD2	LD3	LD4	LD5	LD6
**PC1**	1.13	-0.27	0.30	-0.23	-0.04	0.12
**PC2**	1.21	0.51	-0.40	0.27	-0.04	-0.31
**PC3**	-0.43	0.39	1.18	0.25	0.09	-0.25
**PC4**	0.22	-1.16	0.10	0.44	-0.11	-0.42
**PC5**	0.31	-0.11	-0.10	0.32	1.00	0.21
**PC6**	0.20	0.02	0.17	0.91	-0.38	0.78
**n.pulses**	-0.01	-0.25	0.93	-0.16	0.23	-0.10
**drum_duration**	0.68	0.24	0.70	-0.19	0.31	-0.10
**pulseRateMedian**	-0.46	-1.12	0.35	0.16	-0.34	-0.07
**pulseRateDeviation**	-0.16	0.14	-0.20	-0.04	0.82	0.12
**interval_min**	0.03	1.10	-0.21	0.01	0.17	-0.04
**interval_max**	1.23	0.41	0.18	-0.21	-0.29	-0.17
**interv1_intervMax**	-1.16	-0.20	-0.40	0.00	0.18	0.15
**intervL_intervMax**	-1.06	-0.03	0.19	0.38	0.29	-0.06
**interval_slope_lm2_ordre_coefa**	0.03	0.19	-0.46	-0.06	-0.45	0.23
**interval_nPVI**	1.64	-0.31	-0.18	0.05	-0.06	-0.01
**MeanAcc**	1.50	-0.51	-0.42	0.04	-0.04	0.05
**drum_peak_F**	0.24	0.06	0.51	0.65	-0.19	0.55
**Amp_pulses_min**	0.07	-0.05	-0.41	0.36	-0.02	-0.50
**Amp_pulses_first**	0.32	-0.50	-0.77	-0.30	-0.02	-0.50
**Amp_pulses_last**	0.12	0.18	0.23	0.69	-0.15	-0.26
**Amp_pulses_Q25**	-0.13	0.26	-0.06	0.51	0.10	-0.39
**Amp_pulses_Q75**	0.26	0.34	-0.60	0.34	0.19	0.03
**Amp_pulses_IQR**	0.35	-0.08	-0.38	-0.40	0.01	0.51
**nPAVI**	0.93	-0.43	0.37	-0.09	0.15	0.06
**Amp_slope_lm2_ordre_coefa**	-0.10	-0.37	0.62	0.07	0.10	-0.22
**n_seq**	1.52	-0.23	0.00	0.06	0.20	-0.08
**interval_seq_median**	-0.61	0.55	0.41	-0.44	-0.47	-0.33
**Explained variance**	57.94%	17.41%	13.52%	4.46%	3.80%	2.87%
**Cumulative explained variance**	**57.94%**	**75.35%**	**88.87%**	**93.33%**	**97.13%**	**100.00%**

Supplementary Table 12. Loading scores of the 6 principal components (PCs), and by extension of the 22 acoustic variables, on the first 6 Linear Discriminants (LDs) resulting from the Discriminant Function Analysis carried out on the 6 PCs. Individual and cumulative variance explained by these LDs are indicated in the lower lines of the table.

	Playback-PC1	Playback-PC2
**Latency to drum**	-0.13	0.68
**Number of drums**	0.05	-0.68
**Latency to scream**	-0.45	0.15
**Number of screams**	0.46	0.04
**Maximum approach**	0.52	0.16
**Latency to Maximum approachApproach**	-0.55	-0.17
**Explained variance**	44.18%	26.77%
**Cumulative explained variance**	**44.18%**	**70.95%**

Supplementary Table 13. Loading scores of the 6 behavioral variables on the first 2 principal components (Playback-PC1, Playback-PC2) resulting from the Principal Component Analysis carried out on these variables. Individual and cumulative variance explained by these PCs are indicated in the lower lines of the table. These scores were obtained from the first experiment and used for calculating scores in the second experiment to standardize the interpretation of behavioral responses across experiments.

Supplementary references

[1] Miranda B, Bürgi M. Les pics – habitants exigeants des forêts. *Notice pour le praticien* **40**, (2005).

[2] Gorman G. *Woodpeckers of the World: The Complete Guide*. Christopher Helm (2014).

[3] Askins RA. Foraging Ecology of Temperate-Zone and Tropical Woodpeckers. *Ecology* **64**, 945-956 (1983).

[4] Styring AR, Hussin MZb. Effects of logging on woodpeckers in a Malaysian rain forest: the relationship between resource availability and woodpecker abundance. *J Trop Ecol* **20**, 495-504 (2004).

[5] ONF report: Etat des lieux et diagnostic pour le plan de gestion de la Réserve naturelle des Nouragues 2017-2021.
