# Supplementary material for: Evolution of communication signals and information during species radiation
Source: Nat Commun. 2020 Oct 2;11:4970. doi: 10.1038/s41467-020-18772-3 (PMC7532446; doi:10.1038/s41467-020-18772-3)
Supplement: Supplementary file 3 — Reporting Summary [file 41467_2020_18772_MOESM3_ESM.pdf]

nature research

Corresponding author(s): Maxime Garcia

Last updated by author(s): Aug 27, 2020

Reporting Summary

Nature Research wishes to improve the reproducibility of the work that we publish. This form provides structure for consistency and transparency in reporting. For further information on Nature Research policies, see our Editorial Policies and the Editorial Policy Checklist.

Statistics

For all statistical analyses, confirm that the following items are present in the figure legend, table legend, main text, or Methods section.

n/a	Confirmed	
[ ]	[x]	The exact sample size (n) for each experimental group/condition, given as a discrete number and unit of measurement
[ ]	[x]	A statement on whether measurements were taken from distinct samples or whether the same sample was measured repeatedly
[ ]	[x]	The statistical test(s) used AND whether they are one- or two-sided<br>*Only common tests should be described solely by name; describe more complex techniques in the Methods section.*
[x]	[ ]	A description of all covariates tested
[ ]	[x]	A description of any assumptions or corrections, such as tests of normality and adjustment for multiple comparisons
[ ]	[x]	A full description of the statistical parameters including central tendency (e.g. means) or other basic estimates (e.g. regression coefficient) AND variation (e.g. standard deviation) or associated estimates of uncertainty (e.g. confidence intervals)
[ ]	[x]	For null hypothesis testing, the test statistic (e.g. F, t, r) with confidence intervals, effect sizes, degrees of freedom and P value noted<br>*Give P values as exact values whenever suitable.*
[ ]	[x]	For Bayesian analysis, information on the choice of priors and Markov chain Monte Carlo settings
[x]	[ ]	For hierarchical and complex designs, identification of the appropriate level for tests and full reporting of outcomes
[x]	[ ]	Estimates of effect sizes (e.g. Cohen's d, Pearson's r), indicating how they were calculated

Our web collection on statistics for biologists contains articles on many of the points above.

Software and code

Policy information about availability of computer code

Data collection	No software was used for data collection

Data analysis

Roesch, A. & Schmidbauer, H. WaveletComp: Computational Wavelet Analysis. R package version 1.1. (2018).
Sueur, J., AubORin, T. & Simonis, C. Seewave: a free modular tool for sound analysis and synthesis. Bioacoustics. 18, 213-226 (2008). R package version 2.1.0.
Charrad, M., Ghazzali, N., Boiteau, V. & Niknafs, A. NbClust: An R package for determining the relevant number of clusters in a data set. Journal of Statistical Software 61, 1-36 (2014). R package version 3.0.
Paradis, E. & Schliep, K. ape 5.0: an environment for modern phylogenetics and evolutionary analyses in R. Bioinformatics 35, 526-528 (2018).
Revell, L. J. phytools: An R package for phylogenetic comparative biology (and other things). Methods Ecol. Evol. 3, 217-223 (2012). R package version 0.6-99.
Luke J. H., Weir, J. T., Chad D Brock, C. B., Glor, R. E. & Challenger, W. GEIGER: investigating evolutionary radiations. Bioinformatics 24, 129-131 (2008). R package version 2.0.6.1.
Praat: doing phonetics by computer [Computer program]. v. Version 6.0.30 (University of Amsterdam, The Netherlands, Retrieved from http://www.praat.org/, 2017).
V. Lenth, R. Least-Squares Means: The R Package lsmeans. Journal of Statistical Software 69, 1-33. (2016). R package version 2.27-62.
Bates, D., Maechler, M., Bolker, B. & Walker, S. Fitting Linear Mixed-Effects Models Using lme4. Journal of Statistical Software 67, 1-48 (2015). R package version 1.1-14.
Liaw, A. & Wiener, M. Classification and Regression by randomForest. R News 2(3), 18-22. (2002). R package version 4.6-14.

Custom data analyses and simulation R scripts can be downloaded from the GitHub repository: https://github.com/garciamaxime/DrummingEvolution_SourceData_SourceCodes

For manuscripts utilizing custom algorithms or software that are central to the research but not yet described in published literature, software must be made available to editors and reviewers. We strongly encourage code deposition in a community repository (e.g. GitHub). See the Nature Research guidelines for submitting code & software for further information.

Data

Policy information about availability of data

All manuscripts must include a data availability statement. This statement should provide the following information, where applicable:

- Accession codes, unique identifiers, or web links for publicly available datasets
- A list of figures that have associated raw data
- A description of any restrictions on data availability

All the data are available with the paper and its supplementary files.

Field-specific reporting

Please select the one below that is the best fit for your research. If you are not sure, read the appropriate sections before making your selection.

☐ Life sciences ☐ Behavioural & social sciences ☒ Ecological, evolutionary & environmental sciences

For a reference copy of the document with all sections, see nature.com/documents/nr-reporting-summary-flat.pdf

Ecological, evolutionary & environmental sciences study design

All studies must disclose on these points even when the disclosure is negative.

Study description

This study investigates the evolution of woodpecker drumming, both in terms of acoustic structure and its associated species-specific information content. In-depth acoustic analyses were used to characterize drumming patterns; phylogenetic analyses were used to evaluate the association between structure or information content across woodpecker clades. Field acoustic playback experiments and investigation of natural communities were used to assess the discriminative potential between different species-specific drumming types.

Research sample

Our study relies on drumming recordings from woodpecker species. Recordings belonged to adult individual, where sex was often unspecified. The recorded samples were available from online sound libraries (Xeno-Canto - https://www.xeno-canto.org - and Macaulay library - https:// www.macaulaylibrary.org). The sample of species for which data was collected is representative of the phylogenetic diversity found in the woodpecker family.

Sampling strategy

Sound analysis: we collected a pool of over 2000 audio tracks from online sound archives (Xeno-Canto - https://www.xeno-canto.org - and Macaulay libraries - https://www.macaulaylibrary.org). We assessed the sound quality of these audio tracks by listening and though visual inspection of sound spectrograms. To capture intra-specific variation, we limited audio extraction to one drum per audio track (which also avoided pseudoreplication) and only included species for which at least 3 high-quality drums could be extracted. Aiming for 3 as the minimum number of files per species aimed at being representative of the intra-specific variability found across drumming signals. We therefore retained 736 high-quality drums suitable for further analyses. These drums were distributed among 92 species (out of the 217 recognized species of woodpeckers and 22 genera), providing a representative sampling of the phylogenetic diversity found in this family.
Morphological measurements were made on specimens from the Muséum national d'Histoire naturelle (MNHN, Paris, France) and the Natural History Museum (NHM, Tring, UK). No sample size calculation was performed, and sample size was chosen to match the minimum number of samples collected for audio data.
Playback experiments: we tested 6 different individuals in each condition (total of 48 individuals). Sampling was carried out based on a trade-off between availability among the wild bird population and obtaining a sufficient statistical power.

Data collection	All data collection was carried out by MG, including extraction of audio tracks from online sound archives, field behavioral data and morphological measurements. Sampling of audio data was made based on sample availability. Online audio recording were made using adigital recorder connected to a handheld microphone.<br>Field data collection involved:<br>- a Sennheiser ME67 microphone mounted on a tripod and connected to a digital recorder (Zoom H4N, 44.1 kHz, 16 bit) for audio recordings<br>- an Anchor Megavox loudspeaker placed at about 1 to 1.5m from ground level and connected to an Edirol R-09 for playback experiments<br><br>Morphological measurements was made at the Museum National d'Histoire Naturelle, Paris, France and at the National history museum, tring, UK. Lead author MG had acces to all bird collections and collected measurements from 3 specimens per species to match the minimum number of samples collected for audio data. Measurements werecollected with an Ecotone ornithological ruler (for wing chord), and using digital calipers for beak and tarsus measurements; ± 0.02 mm accuracy for < 10 mm measurements and ± 0.03 mm accuracy for > 10 mm measurements).
Timing and spatial scale	Playback experiments were carried out on wild individuals around Saint-Etienne (spanning a range of 50km around Saint-Etienne), France, during this species' breeding season (February-April 2017).<br>Data collection from online libraries was made between November 2016 and March 2017, comprising species recorded from all around the world, and only limited by sample availability, in order to get a sampling maximizing spatial range.
Data exclusions	No data were excluded from the analyses.
Reproducibility	All codes used in our analyses have been verified multiple times to avoid reproducibility errors.
Randomization	We used a randomized order design for playback experiments. Samples from online libraries were allocated to a 'group' simply by referring to their species identity and included as many recording as possibly available with a minimum of 3 samples per species).
Blinding	This is only applicable to field playback experiments. In order to minimize bird disturbance and only one experimenter conducted the playback experiments. Because visual confirmation of the birds was not possible, acoustic recordings and written notes were the only methods used to document the birds responses, and blind coding by another author (who had not conducted the experiments) would have led to biased behavioral assessment (as direct feedback during the experiments was crucial for this). Therefore, no blinding was carried out for our analyses.

Did the study involve field work? ☒ Yes ☐ No

Field work, collection and transport

Field conditions	Field playback experiments were performed in February-April 2017 (end of winter - beginning of spring), which corresponds to the Great-spotted woodpecker's breeding season. Experiments were carried out in the great-spotted woodpecker's habitat (mixed coniferous and deciduous forests), on mornings where wind conditions were low and the weather ranged from cloudy to sunny (but never on rainy days; besides, temperature ranged 5 to 15 degrees Celsius).
Location	Playback experiments were carried out on wild great-spotted woodpeckers around Saint-Etienne, France, during the breeding season (February-April 2017). The research area ranged 50km around Saint-Etienne (Latitude 45.433 N; Longitude:4.39 E) and 300m-1000m elevation.
Access & import/export	No import/export was made on the birds tested. The experimenter parked the car (used to travel from one study site to the other) and walked to the great-spotted woodpeckers' territories in order to generate minimal disturbance. All work is in compliance with national and international laws and did not require specific permits.
Disturbance	Minimal disturbance. Woodpeckers during the breeding season are often exposed to conspecifics drumming in their vicinity. Our playback experiments simulated the presence of one such conspecific. The experimenter was hidden underneath a camouflage net and did not interfere with the focal individual's response.

Reporting for specific materials, systems and methods

We require information from authors about some types of materials, experimental systems and methods used in many studies. Here, indicate whether each material, system or method listed is relevant to your study. If you are not sure if a list item applies to your research, read the appropriate section before selecting a response.

Materials & experimental systems

n/a	Involved in the study
☒	☐ Antibodies
☒	☐ Eukaryotic cell lines
☒	☐ Palaeontology and archaeology
☐	☒ Animals and other organisms
☒	☐ Human research participants
☒	☐ Clinical data
☒	☐ Dual use research of concern

Methods

n/a	Involved in the study
☒	☐ ChIP-seq
☒	☐ Flow cytometry
☒	☐ MRI-based neuroimaging

Animals and other organisms

Policy information about studies involving animals; ARRIVE guidelines recommended for reporting animal research

Laboratory animals	None
Wild animals	Wild great spotted woodpeckers (Dendrocopos major; adults, undetermined sex) were localized by their drumming behavior. Playbacks started at about 50m from where the experimenter last saw or heard the focal individual. Playback intensity was calibrated and kept at about 80 dB measured 1m away from the speaker, similar to the natural drumming intensity. Behavioral data collection started when the first drum of the stimuli sequence was broadcasted and lasted 10minutes from that moment on. No capture or bird handling was ever performed.
Field-collected samples	Only acoustic recordings were collected for the scope of this study.
Ethics oversight	All experiments were performed in accordance with relevant guidelines and regulations including French national guidelines, permits and regulations regarding animal care and experimental use (approval no. D42-218-0901, ENES lab agreement, Direction Départementale de la Protection des Populations, Préfecture du Rhône)

Note that full information on the approval of the study protocol must also be provided in the manuscript.
