# Supplementary material for: Evolution of communication signals and information during species radiation
Source: Nat Commun. 2020 Oct 2;11:4970. doi: 10.1038/s41467-020-18772-3 (PMC7532446; doi:10.1038/s41467-020-18772-3)
Supplement: Supplementary file 4 — Description of Additional Supplementary Files [file 41467_2020_18772_MOESM4_ESM.pdf]

Description of Additional Supplementary Files

File Name: Supplementary Data 1
Description: Raw acoustic data extracted for each of the 736 drumming recordings included in this study

File Name: Supplementary Data 2
Description: Raw morphological measurements (median values) and geographical data collected for each species in the study
